# CD24: a marker of granulosa cell subpopulation and a mediator of ovulation

**DOI:** 10.1038/s41419-019-1995-1

**Published:** 2019-10-17

**Authors:** Jun-peng Dong, Zhi-hui Dai, Zhong-xin Jiang, Yi He, Liang Wang, Qiu-ying Liao, Ning-xia Sun, Yi-ning Wang, Shu-han Sun, Wei Lin, Wen Li, Fu Yang

**Affiliations:** 1 The Department of Medical Genetics, Second Military Medical University, 200433 Shanghai, China; 2 The Center of Reproductive Medicine, Shanghai Changzheng Hospital, Second Military Medical University, 200003 Shanghai, China; 3 Translational Genomics Research Institute, Molecular Medicine Division, Phoenix, AZ USA; 4 Hunan Provincial Key Lab of Emergency and Critical Care, Hunan People’s Hospital, Changsha, Hunan China; 5 Shanghai Key Laboratory of Cell Engineering (14DZ2272300), Shanghai, People’s Republic of China

**Keywords:** Predictive markers, Endocrine reproductive disorders

## Abstract

Granulosa cells (GCs) play a critical role in driving the formation of ovarian follicles and building the cumulus-oocyte complex surrounding the ovum. We are particularly interested in assessing oocyte quality by examining the detailed gene expression profiles of human cumulus single cells. Using single-cell RNAseq techniques, we extensively investigated the single-cell transcriptomes of the cumulus GC populations from two women with normal ovarian function. This allowed us to elucidate the endogenous heterogeneity of GCs by uncovering the hidden GC subpopulation. The subsequent validation results suggest that CD24(+) GCs are essential for triggering ovulation. Treatment with human chorionic gonadotropin (hCG) significantly increases the expression of CD24 in GCs. CD24 in cultured human GCs is associated with hCG-induced upregulation of prostaglandin synthase (ARK1C1, PTGS2, PTGES, and PLA2G4A) and prostaglandin transporter (SLCO2A1 and ABCC4) expression, through supporting the EGFR-ERK1/2 pathway. In addition, it was observed that the fraction of CD24(+) cumulus GCs decreases in PCOS patients compared to that of controls. Altogether, the results support the finding that CD24 is an important mediator of ovulation and that it may also be used for therapeutic target of ovulatory disorders.

## Introduction

In humans, ovarian folliculogenesis is a complex physiological process that underlies the health of the subsequent embryo and offspring^1,2^. Oocyte quality is central to female fertility, and oocyte developmental competence is a key rate-limiting factor in the modern practice of assisted reproductive technology^3^. Oocyte maturation and ovulation involve multiple intertwined intra-ovarian and endocrine processes^4,5^. Bidirectional somatic granulosa cell-oocyte signaling plays an important role in determining an oocyte’s developmental fate. Oocyte-derived proteins of the transforming growth factor superfamily (i.e., bone morphogenetic protein 15 [BMP15] and growth differentiation factor 9 [GDF9]) interact with surrounding granulosa cells (GCs), which subsequently produce paracrine factors, i.e., glial-derived neurotrophic factor (GDNF) and prostaglandin metabolites, to mediate oocyte maturation, GC proliferation, and differentiation^6–9^.

Considering the bidirectional GC-oocyte signaling, it has been proposed to assess the competence of the oocyte and embryonic development potential by assessing the quality of the GCs. Previous studies indicate that increasing cumulus GC apoptosis is accompanied by impaired oocyte competence and reduced pregnancy outcomes^10,11^. Cumulus GCs obtained from high-quality oocytes have greater expression levels of HAS2, GREM1, and PTGS2 than cumulus GCs from poor-quality oocytes^12,13^. Some investigators have proposed a noninvasive test by scanning gene expression profiles of human cumulus GCs^14,15^. Nonetheless, which genes in GCs are related to and thus able to serve as biomarkers of oocyte competence or embryo potential remains an interesting but unsolved problem.

Compared with healthy ovulations, oocyte competence and embryo potential are oftentimes altered in women with polycystic ovary syndrome (PCOS)^16^. GC dysfunction may contribute to the aberrant folliculogenesis observed in PCOS^17,18^. Altered epigenetics and gene expression have been shown to contribute to the aberrant function of GCs from PCOS patients^18,19^. These studies uncover the molecular alterations associated with the progress of follicle development. Nonetheless, the authors did not address the contributions of the putative GC subsets or individual cells, nor did they account for the cellular heterogeneity of GCs. This leaves a major gap in our understanding of the molecular mechanisms controlling follicle development.

Single-cell RNA sequencing (scRNA-seq) is a new technique that enables us to dissect heterogeneous multiple-cellular tissues such as cancer cells^20^ and immunocytes^21^. In this study, we performed scRNA-seq and analyzed hundreds of GCs from two healthy women. It was found that CD24(+) GCs triggered ovulation and that the fraction of CD24(+) cumulus GCs decreased in PCOS patients. This study allows us to elucidate the endogenous heterogeneity of GCs in the human oocyte-cumulus complex and uncover novel GC subpopulations with specific markers.

## Results

### Identification of the hidden CD24(+) GC subpopulations

**Fig. 1:**
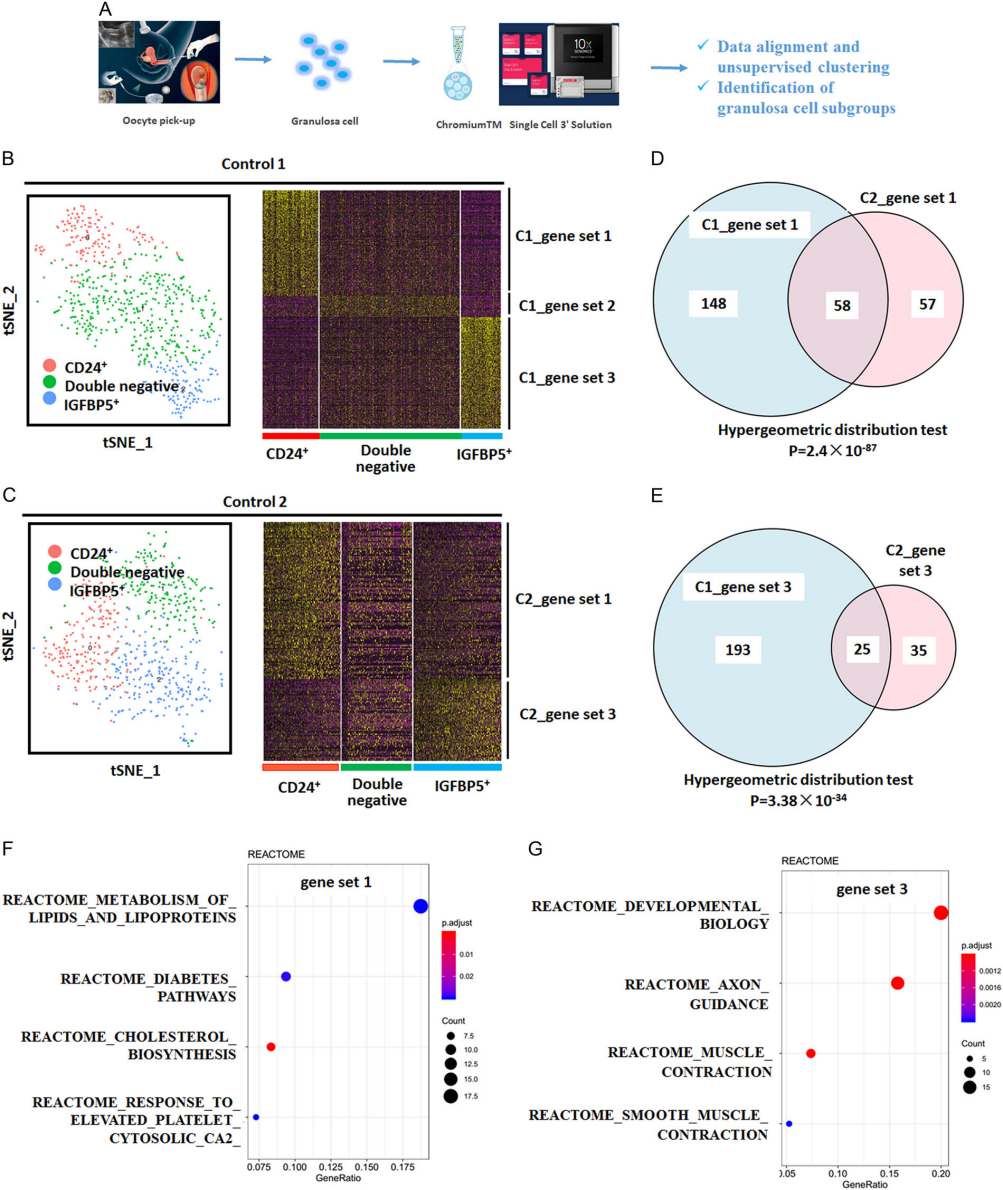
Single-cell transcriptomes recapitulate the major cumulus GCs. **a** Workflow for obtaining and analyzing scRNA-Seq data from human cumulus GCs. **b** Spectral t-SNE plots of all cumulus GCs analyzed from the first woman with normal ovarian function (control 1, C1), annotated by cell-type identity (left). Hierarchical clustering of cells (column) using the differentially expressed gene (rows) sets that distinguish each subpopulation from the remaining cells in C1. Cells are classified into three clusters (bottom bars) (Right). **c** Spectral t-SNE plots of all cumulus GCs analyzed from the second woman with normal ovarian function (control 2, C2), annotated by cell-type identity (left). Hierarchical clustering of cells (column) using the differentially expressed gene (rows) sets that distinguish each subpopulation from the remaining cells in C2. Cells are classified into three clusters (lower sidebar) (Right). **d** Venn diagram analysis and hypergeometric test between gene set 1 of C1 and gene set 1 of C2. **e** Venn diagram analysis and hypergeometric test between gene set 3 of C1 and gene set 3 of C2. Results of pathway-based analysis of gene set 1 (combined gene set 1 of C1 and C2, F) and gene set 3 (combined gene set 3 of C1 and C2, G) showing significant pathways in REACTOME

We performed scRNA-seq on freshly collected GC samples from two healthy women (control 1 and control 2) using a 10x single-cell RNAseq platform (Fig. 1a). The high-dimension scRNA-seq gene expression data were transformed and visualized using dimensional reduction and unsupervised clustering algorithms. We identified three reproducible clusters of cells visualized on the cell layouts based on the t-SNE algorithm (Fig. 1b, c). The three signature gene sets (gene sets 1-3, Supplementary Tables 1–3) were identified to distinguish each subpopulation from the other cells in both samples (Fig. 1b, c). To test the concordance between gene set 1 of control 1 (C1) and gene set 1 of control 2 (C2), we use a Venn diagram for illustration and perform a hypergeometric test on the gene sets. A total of 58 common genes were identified in gene set 1 of C1 and C2 (Fig. 1d). The *p*-value of the hypergeometric test is 2.4 × 10^−87^. Also, in gene set 3 of C1 and C2, we identified 25 common genes (Fig. 1e). The *p*-value is 3.38 × 10^−34^. All of these results indicate the concordance of clusters in both samples and reproducible subpopulations.

We cross-compared the gene sets defined in Reactome annotations^22^ and tried to correlate the annotated gene sets to the signature genes of the putative subpopulations (Fig. 1f, g). For gene set 1 (combined from both C1 and C2), we had two hits involved in steroid biosynthesis, i.e., metabolism of lipids and lipoproteins and cholesterol biosynthesis, that are highly expressed in the first GC cluster (Fig. 1f). The hits indicate that lipid metabolism and steroid biosynthesis may be active in this GC subpopulation. The genes involved in the significant pathways are listed in Supplementary Table 4.

**Fig. 2:**
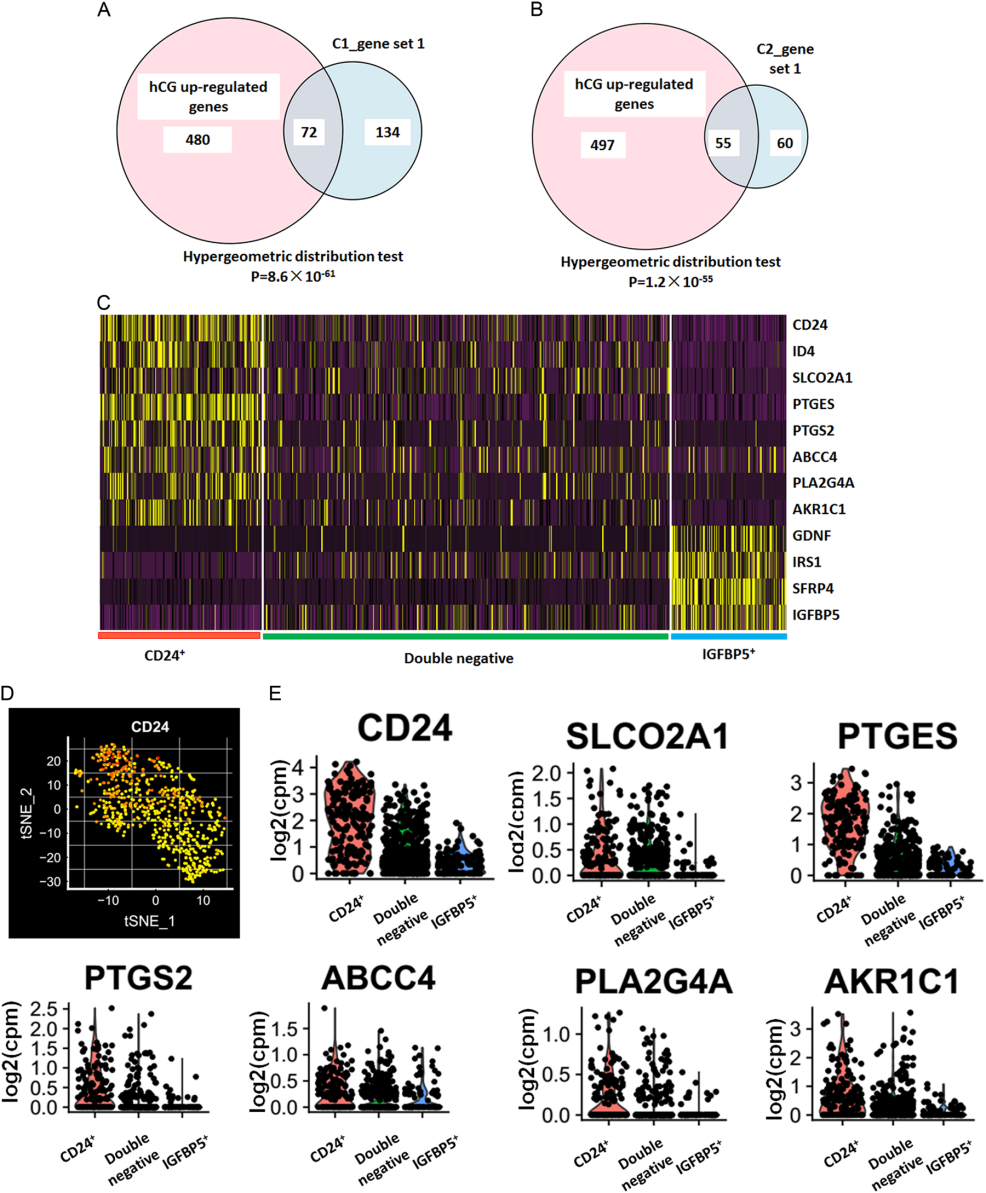
Expression levels of known key genes of cumulus GCs. **a** Venn diagram analysis and hypergeometric test between gene set 1 of C1 and hCG upregulated genes. **b** Venn diagram analysis and hypergeometric test between gene set 1 of C2 and hCG upregulated genes. **c** Heatmap of the mean expression of known key genes within each subpopulation in C1. Cells are classified into three clusters (bottom bars). **d** t-SNE plots for expression of CD24 in C1. Each yellow point represents a single cell. The color gradient in the t-SNE plot represents the relative expression level of CD24 in a cell across the whole population and subpopulations: (light red) low; (dark red) high. **e** Violin plots for expression of CD24, prostaglandin synthases (ARK1C1, PTGS2, PTGES, and PLA2G4A) and prostaglandin transporters (SLCO2A1 and ABCC4) in C1. Each point represents a single cell

To identify the GC subpopulation involved in the human ovulatory cascade and final oocyte maturation, we reviewed the scRNA-seq data based on the results of a previous comparison of GC transcriptomes before and after ovulation triggering using microarray^23^. Nine pairs of transcriptomes from bulk GCs before and after recombinant human chorionic gonadotrophin (hCG) administration were compared. A total of 552 genes were found to be upregulated (hCG upregulated genes) and 614 genes were downregulated (hCG downregulated genes) by selecting differential genes with a *p*-value cutoff < 0.0001 (paired *t*-test, with FDR < 0.0012) and fold difference > 2. Using a Venn diagram and hypergeometric test, we found that the corresponding gene set 1 of both samples is enriched in recombinant human chorionic gonadotrophin (rhCG) upregulated sets (Fig. 2a, b). We further examined the transcriptional dynamics in the subpopulations based on the expression of previously described genes correlated to the human ovulatory cascade and oocyte maturation including CD24^23^, ID4^24^, PLA2G4A^25^, PTGES^25^, PTGS2^25^, ARK1C1^25^, SLCO2A1^9^, ABCC4^25^, SFRP4^26^, GDNF^27^, IRS1^28^, and IGFBP5^29^ (Fig. 2c–e and Supplementary Fig. S1). It is interesting to find that hCG upregulated genes, including CD24 (a heavily glycosylated mucin-type glycosylphosphatidylinositol-anchored cell surface molecule), prostaglandin synthases (AKR1C1, PLA2G4A, PTGES, and PTGS2), and prostaglandin transporters (SLCO2A1 and ABCC4), were highly expressed in one GC cluster (Fig. 2c–e and Supplementary Fig. S1). Considering the specific overexpression of CD24 in this GC subpopulation, we named it CD24(+) GCs. Meanwhile, SFRP4, GDNF, IRS1, and IGFBP5 are highly expressed in another GC cluster (Fig. 2c and Supplementary Fig. S1a). We defined IGFBP5(+) GCs and double-negative GCs based on their CD24 and IGFBP5 signal.

**Fig. 3:**
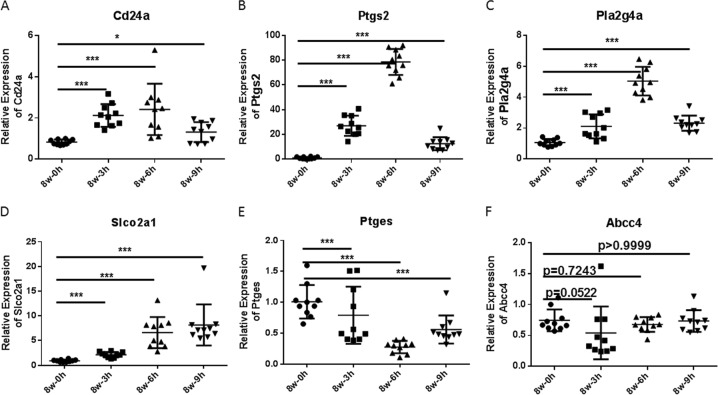
mRNA expression pattern in superovulated mice. Whole cumulus GC RNA was isolated from 8-week-old female mice primed with 5 U of PMSG for 48 h at the designated time points after administration of 5 U of hCG. Expression of Cd24a (**a**), Ptgs2 (**b**), Pla2g4a (**c**), Slco2a1 (**d**), Ptges (**e**), and Abcc4 (**f**) was quantified by real-time PCR and normalized to GAPDH expression (*n* = 10 mice per group). Data are means ± SEMs. The Wilcoxon signed-rank test was used. *p < 0.05 and ***p < 0.001

To further explore the in vivo regulation of granulosa cellular CD24, AKR1C1, PLA2G4A, PTGES, PTGS2, SLCO2A1, and ABCC4 expression, we used the pregnant mare serum gonadotropin (PMSG)-primed/hCG-triggered immature mouse superovulation model. As shown in Fig. 3, whole mouse cumulus GC Cd24a, Pla2g4a, Ptgs2, and Slco2a1 transcripts increased after the administration of hCG. These results show the time-dependent induction of Cd24a, Pla2g4a, Ptgs2, and Slco2a1 in mouse periovulatory follicles. The expression of Ptges decreased after the administration of hCG to a minimum noted 6 h later. Whole mouse cumulus GC Abcc4 transcripts did not change after hCG administration, and the expression of Akr1c1 could not be detected using real-time PCR. Considering the important regulatory roles of prostaglandin synthases (PLA2G4A, PTGES and PTGS2) and prostaglandin transporters (SLCO2A1) in the human ovulatory follicles^9,30^, we selected the CD24(+) GCs as subjects for further research.

### CD24 mediates the EGFR and EGFR-ERK1/2 pathways in GCs

CD24 expression in cancer is related to tumor aggressiveness, especially cell invasion^31^ and cancer cell stemness^32^. However, the massive upregulation of CD24 expression following rhCG exposure in GCs is unexpected (Fig. 3a). We therefore extensively investigated the role of CD24 in ovulation.

**Fig. 4:**
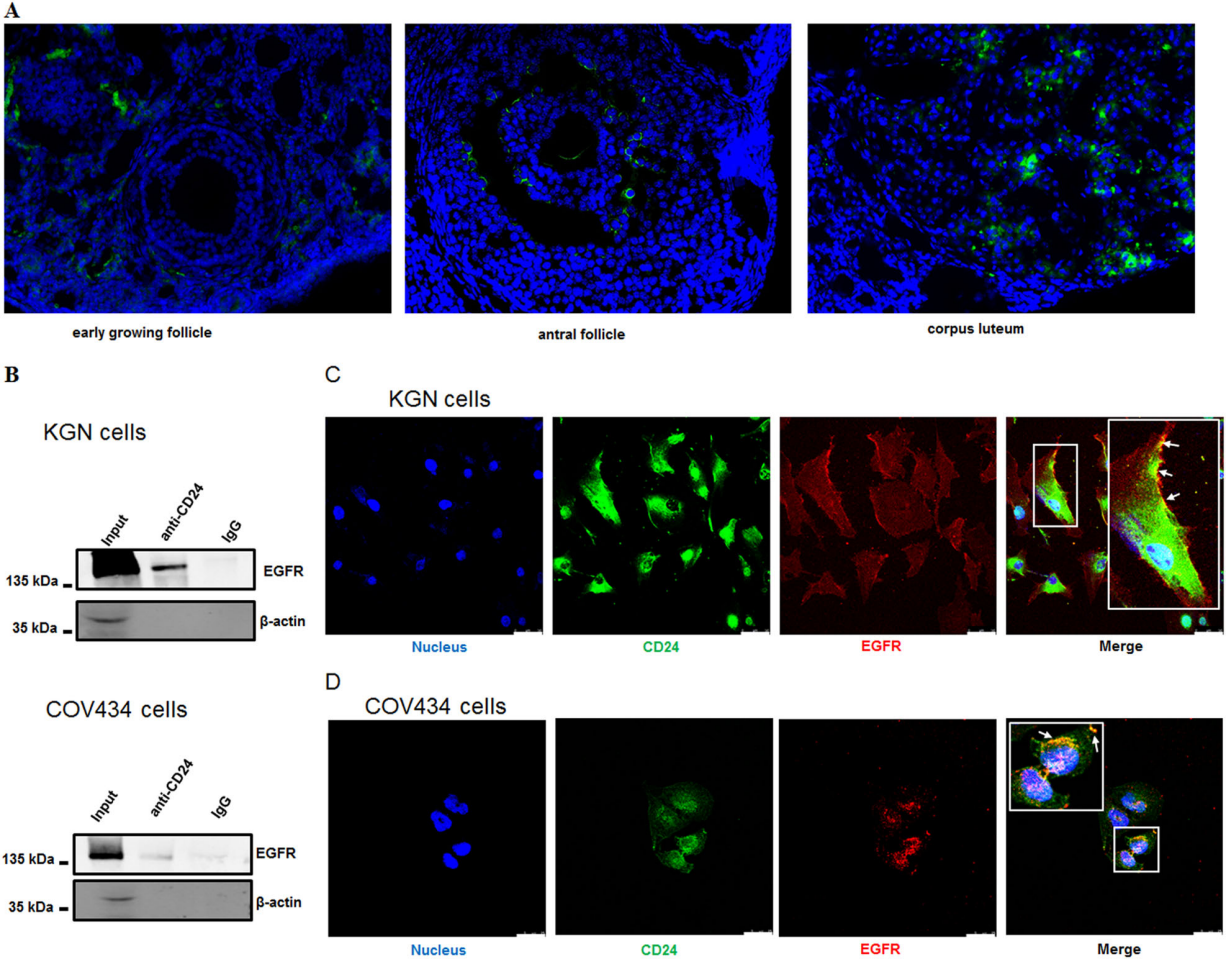
CD24 forms complexes with EGFR. **a** Immunofluorescence staining for Cd24a in a mouse early-growing follicle (left), antral follicle (middle) and corpus luteum (right). Χ200. **b** Co-immunoprecipitation of EGFR by CD24 was determined. KGN cells (up) or COV434 cells (down) were immunoprecipitated with anti-CD24 antibody, followed by western blotting assays for EGFR. The second panel shows β-actin bands in samples of input and immunoprecipitation. Representative micrographs of KGN (**c**) and COV434 (**d**) cells stained for CD24 (green) and EGFR expression (red) by immunofluorescence. The arrow shows colocalization of CD24 and EGFR. Scale bar, 50 μm. *n* = 3 for all experiments

To examine the expression pattern of the Cd24a protein in the ovary, we subjected histological sections of the ovaries of normally cycling mice to the immunofluorescence staining. As shown in Fig. 4a, the expression of the Cd24a protein in GCs of early-growing follicles is negligible. In contrast, the expression of the Cd24a protein in GCs of antral follicles and the corpus luteum remarkably increase (Fig. 4a). These results indicate that the expression of Cd24a transcripts in GCs may be ovulation-dependent. It is known that acute upregulation of the EGFR pathway is an essential component of the ovulatory cascade as it transmits the luteinizing hormone (LH) signal from the periphery of the follicle to the cumulus-oocyte complex^4^. Previous studies also indicate that CD24 regulated EGFR signaling by inhibiting EGFR internalization and degradation in cancer cells^33,34^. Therefore, we are wondering whether CD24 and EGFR are physically connected in GCs. Using co-immunoprecipitation assays, it is shown that these two proteins can be co-precipitated in KGN and COV434 cells (Fig. 4b). We next investigated whether there is physical interaction between CD24 and EGFR. Notably, CD24 and EGFR showed partial colocalization on the surface of both KGN (Fig. 4c) and COV434 cells (Fig. 4d). These results suggest that CD24 is indeed physically connected to EGFR.

**Fig. 5:**
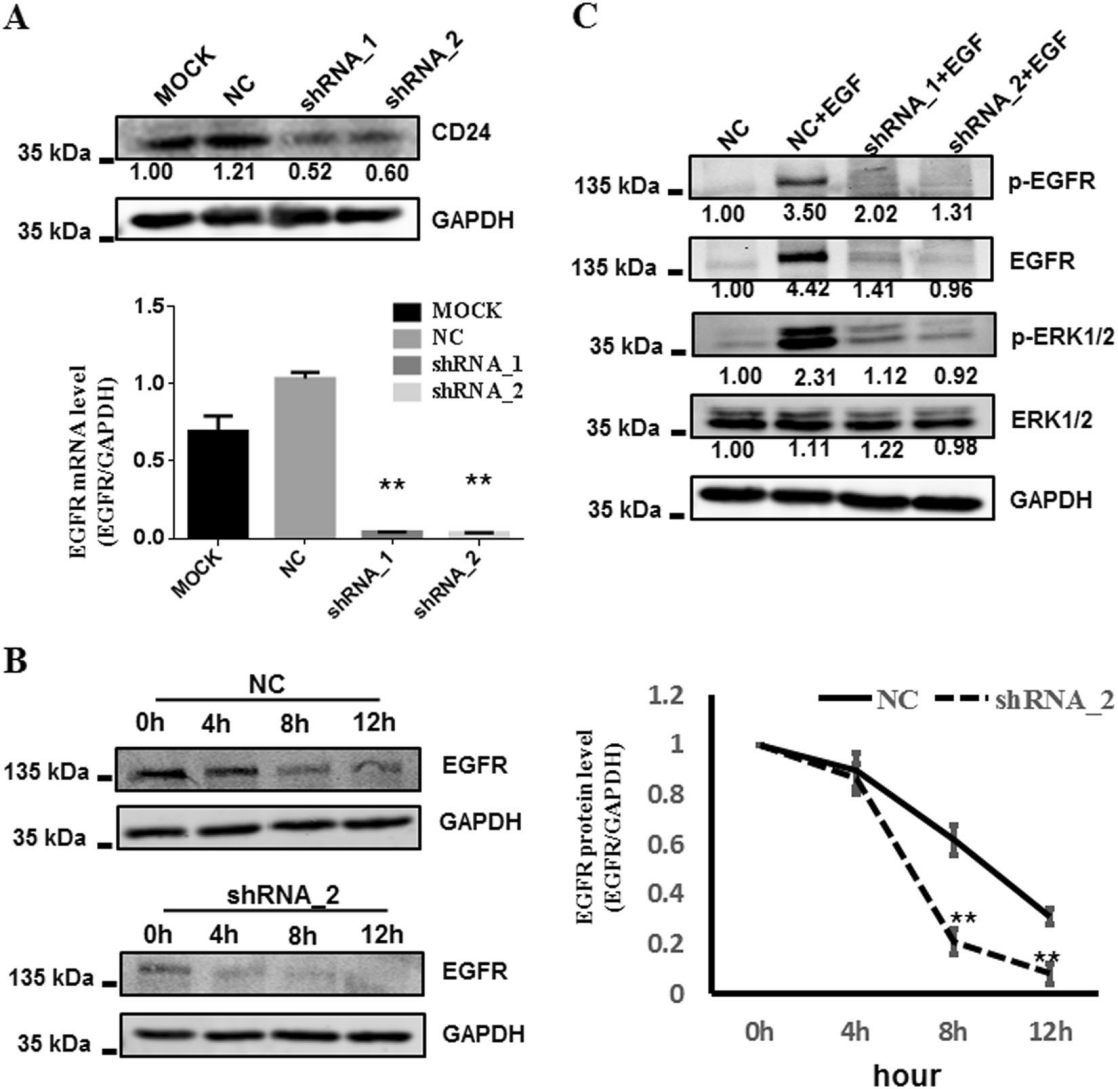
Depletion of CD24 attenuates EGFR/ERK1/2 signaling cascades. **a** CD24 depletion was verified by western blotting (up) and real-time PCR (down). MOCK untreated COV434 cells, NC negative control, COV434 cells infected with shRNA lentiviral particles with noneffective scramble shRNA sequences; shRNA_1 and shRNA_2, COV434 cells infected with shRNA lentiviral particles with CD24 shRNA_1 or CD24 shRNA_2 sequences. **b** The effect of CD24 knockdown on the protein stability of EGFR. COV434 cells infected with control shRNA (NC negative control) or CD24 shRNA (shRNA_2) lentivirus were treated with CHX (20 ug/mL) over the course of 12 h. Loss of CD24 greatly decreased the protein stability of EGFR. **c** The effect of CD24 depletion on EGF-driven ERK1/2 signaling cascades in COV434 cells. Western blot analysis showed that knockdown of CD24 led to the reduction of EGF− induced EGFR/ERK1/2 signaling cascades. Each experiment was performed at least three times

We further analyzed the effect of CD24 on EGFR-related signaling pathways in GCs. The expression of CD24 was suppressed by the shRNA in COV434 cells (Fig. 5a). Cycloheximide (CHX) is the most common laboratory reagent used to inhibit protein synthesis. CHX has been shown to block the elongation phase of eukaryotic translation^33^. Cells infected with the control shRNA or CD24 shRNA lentivirus were both treated with CHX (20 ug/mL) over the course of 12 h. Loss of CD24 greatly reduced the stability of EGFR protein (Fig. 5b). As expected, the levels of phosphorylated EGFR protein (p-EGFR) and its downstream effectors phosphorylated ERK1/2 (p-ERK1/2) were decreased in CD24 shRNA lentivirus-transfected cells compared with the levels in controls (Fig. 5c). Taken together, these observations suggest that CD24 associates with EGFR and supports the EGFR-ERK1/2 pathway in GCs.

### The CD24-EGFR-ERK1/2 pathway increases prostaglandin synthases and prostaglandin transporters

**Fig. 6:**
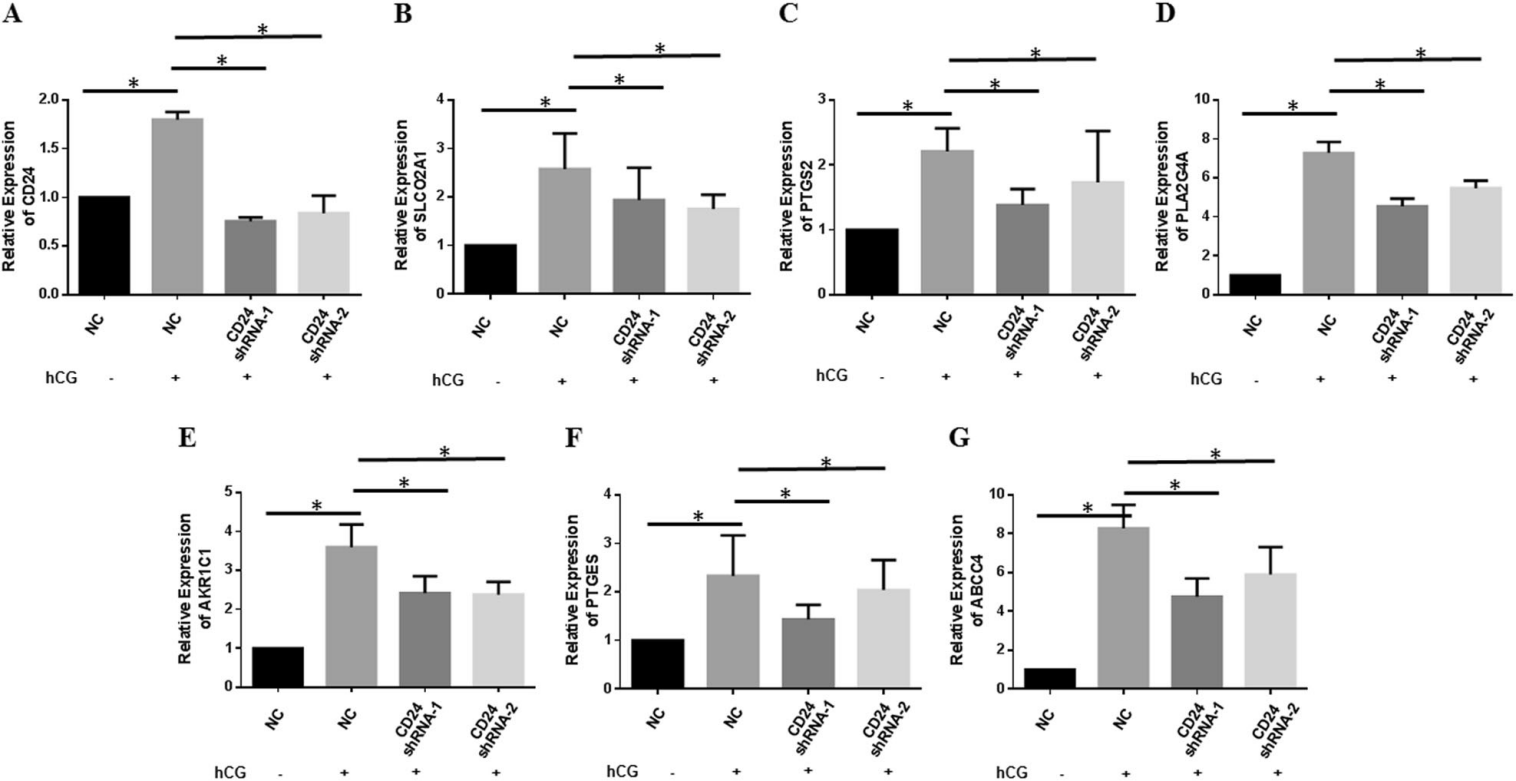
CD24 loss results in decreased expression of prostaglandin synthases and prostaglandin transporters in GCs. GCs aspirated during IVF procedures were initially cultured for 4 days and then infected with CD24 shRNA lentivirus. FSH-pretreated cells (another 48 h) were exposed to hCG (1 U/ml) for 24 h. CD24 (**a**), SLCO2A1 (**b**), PTGS2 (**c**), PLA2G4A (**d**), AKR1C1 (**e**), PTGES (**f**), and ABCC4 (**g**) mRNA expression was quantified by real-time PCR and normalized to GAPDH expression. The results are expressed as the fold change with respect to the control. Data are means ± SEMs of three independent experiments. *p < 0.05

Our results suggests that CD24, prostaglandin synthases (PLA2G4A, PTGES, and PTGS2) and prostaglandin transporters (SLCO2A1 and ABCC4) are highly expressed in CD24(+) GCs (Fig. 2c–e). The expression levels of Cd24a, Pla2g4a, Ptgs2, and Slco2a1 transcripts are consistent after the administration of hCG (Fig. 3). A previous study indicates that the activated EGFR-ERK1/2 pathway is required to induce PTGS2 and SLCO2A1 expression in GCs^9^. Therefore, we further examined the role of CD24 supporting the EGFR-ERK1/2 pathway in the expression of prostaglandin synthases (ARK1C1, PTGS2, PTGES, and PLA2G4A) and prostaglandin transporters (SLCO2A1 and ABCC4). To this end, FSH-pretreated CD24 downregulated primary human cultured GCs (48 h) were incubated with hCG (5 U/ml) for 24 h. As shown in Fig. 6b–g, CD24 shRNA effectively abolishes the hCG-induced upregulation of SLCO2A1 (Fig. 6b), PTGS2 (Fig. 6c), PLA2G4A (Fig. 6d), AKR1C1 (Fig. 6e), PTGES (Fig. 6f), and ABCC4 (Fig. 6g). Further, FSH-pretreated human cultured GCs (48 h) were initially pretreated with or without U0126 [an inhibitor of MEK (mitogen-activated protein kinase kinase), an upstream activator of ERK; 10 mM] for 1 h and then re-incubated with hCG (5 U/ml) for 24 h. Finally, pretreatment with U0126 remarkably inhibits the hCG-mediated upregulation of prostaglandin synthases and prostaglandin transporters (Supplementary Fig. S2). These observations suggest that the CD24-EGFR-ERK1/2 signaling pathway is involved in the hCG-induced upregulation of prostaglandin synthase (ARK1C1, PTGS2, PTGES, and PLA2G4A) and prostaglandin transporter (SLCO2A1 and ABCC4) expression in GCs.

### Decrease of CD24 expression and the downstream effect in GCs of PCOS patients

**Fig. 7:**
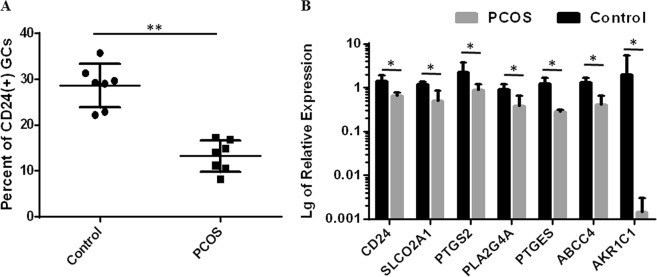
CD24 is downregulated in GCs of PCOS patients. **a** Flow cytometry analysis of CD24(+) GCs from the indicated patients (*n* = 7). **b** Graph showing the levels of CD24, SLCO2A1, PTGS2, PLA2G4A, AKR1C1, PTGES, and ABCC4 in five PCOS patients and five healthy controls. The expression level was detected via real-time PCR and normalized against GAPDH. **p < 0.01, *p < 0.05

PCOS is a complex endocrine condition characterized by oligo/anovulation, high androgen levels, and polycystic ovaries. We speculated that the dysfunction of CD24(+) GCs may contribute to the development of PCOS. Through flow cytometry analysis, we found that the fraction of CD24(+) GC subpopulation decreased significantly in PCOS (*n* = 7) patients compared to the control (*n* = 7) patients (Fig. 7a). We then examined the relative mRNA abundances of prostaglandin synthases and prostaglandin transporters using GCs collected from PCOS (*n* = 5) over the control (*n* = 5) patients who underwent IVF-ET. As shown in Fig. 7b, decreased mRNA abundances of CD24, PTGS2, SLCO2A1, PTGES, ARK1C1, PLA2G4A, and ABCC4 were observed in GCs of PCOS patients compared with those of the control patients. These results suggested that low expression levels of CD24, prostaglandin synthases and prostaglandin transporters in GCs is correlated to the lack of ovulation in PCOS patients and to the development of PCOS.

## Discussion

Cumulus GCs are closely adjacent to the oocyte. Ample results in recent studies have shown the predictive power of some cumulus GC genes for oocyte developmental potential^10–12,15^. As cumulus GCs can be easily collected during the process of intracytoplasmic sperm injection, it is encouraging to characterize the potential biomarkers for prediction of oocyte developmental potential in IVF clinics. In the current study, we acquired single-cell transcriptome data for over 700 cells and repeatedly characterized three subpopulations in two samples. In addition, we used flow cytometry and real-time PCR to validate the findings from our scRNA-seq studies. For the first time, we provide evidence to demonstrate the heterogeneity of cumulus GCs. Moreover, we discussed the markers of cumulus GC subgroups, particularly CD24, for their predictive role in assessment of the potential of oocyte development.

CD24 is present in a variety of cell types in mammalian organisms such as hematopoietic cells and cancer cells. It serves as an important signaling molecule and mediates signal transduction by recruiting Src family protein tyrosine kinases^35^ or associating with EGFR^33,34^. The upregulation of CD24 in GCs was first reported in hGC exposure^23^, which defined CD24 as an ovulation-regulatory gene, though its function remains unclear. We detected the cumulus GC subpopulation to be 20–30% in women with normal ovarian function (Fig. 7a), whereas a significant decrease in this subpopulation is found in PCOS patients. This finding may provide a new clue for PCOS pathogenesis. Second, we uncovered the binding of CD24 and EGFR on the surface of GCs. Given the key roles of the epidermal growth factor network in the regulation of oocyte maturation and ovulation^4^, CD24 might be another player in the oocyte-cumulus complex. As expected, we further discovered that CD24 is associated with EGFR-ERK1/2 pathway activation and the expression elevation of the prostaglandin synthases (ARK1C1, PTGS2, PTGES, and PLA2G4A) as well as the prostaglandin transporters (SLCO2A1 and ABCC4) in GCs. Evidence has shown that prostaglandins are involved in the female reproductive process, especially ovulation^9^. These findings are filling the gap in the understanding of prostaglandin signaling, enhancing the understanding of ovulatory disorders, and facilitating the treatment of infertility or subfertility in women by using nonsteroidal prostaglandin-based therapeutic approaches.

Overexpression of CD24 has been reported in many cancer studies^31,36,37^. CD24 has also been reported to promote cancer cell angiogenesis^33,38^. However, overexpression of CD24 in the corpus luteum is new to us (Fig. 4a). For the first time, the relationships across CD24 expression, vascularization, and angiogenesis characterizing corpus luteum formation are unveiled, which demands extensive studies to understand CD24’s functional role in these processes.

In this study, we demonstrated the power of scRNA-seq in revealing a hidden subpopulation of seemingly homogeneous cells by automatically clustering the overall similar single-cell transcriptional profiles. In the study of the GC population, the reproducibility of the CD24(+) subpopulation in two replicates makes our findings very convincing. In addition, the counterpart CD24(−) subpopulation in GCs is another interesting subpopulation whose role is yet to be explored. Functional genes that are co-expressed in the same population will provide clues to further explore the function of each subpopulation. In the meantime, we are looking forward to including more patients/samples to validate the key molecules discovered at the single-cell resolution.

## Materials and methods

### Human subjects and GC samples

The ethics committee of Changzheng Hospital of the Second Military Medical University approved this study, and informed consent was obtained from all participants. Twelve PCOS patients, diagnosed according to the revised Rotterdam diagnostic criteria for PCOS^39^, and fourteen women with normal ovarian function (serving as controls) seeking in vitro fertilization (IVF)-intracytoplasmic sperm injection (ICSI) treatment (IVF-ICSI) at the Changzheng Hospital of the Second Military Medical University were recruited. The control women with normal ovarian function (control) met the following inclusion criteria: (1) age between 22 and 30; (2) without ovarian morphological abnormalities by ultrasound examination; (3) normal ovarian response; (4) regular menstrual cycles (26–33 days); (5) no signs of hyperandrogenism; (6) BMI > 18 and < 25; and (7) without polycystic ovary morphology. The GnRH antagonist stimulation protocol for controlled ovarian hyperstimulation was performed as previously described^40^. The major anthropometric variables and endocrine parameters of the women are presented in Supplementary Table 5. We performed transvaginal oocyte aspiration with ultrasound guidance under local anesthesia 36 h after injection of hCG. The GCs were obtained from MII-stage cumulus-oocyte complexes (COCs) by follicular aspiration from the women undergoing oocyte retrieval for IVF-ICSI and rinsed in G-MOPS medium (Vitrolife, Sweden). The cells were dispersed by gentle pipetting in 1% hyaluronidase enzyme and washed with G-MOPS. Then, they were transported on ice to the laboratory and resuspended in ice-cold Red Blood Cell Lysis Buffer (Beyotime institute of Biotechnology, China) for 2 min. Next, the cells were washed with PBS and centrifuged at 550 × *g* for 5 min. The isolated GCs were immediately used for scRNA-seq, flow cytometric analysis, cultured or stored at −80 °C, for real-time PCR analysis. For culture of primary GCs, 1 × 10^6^ cells were cultured in a 24-well culture plate in DMEM:F12 (Gibco, USA) supplemented with 10% fetal bovine serum (Gibco, USA) and antibiotics (100 U/ml penicillin, 100 mg/ml streptomycin, and 0.25 mg/L amphotericin B) in a humidified atmosphere of 5% CO2 and 95% air at 37 °C. The average follicular diameter used for the analysis of GCs is 16 mm, and the minimum follicular diameter used to obtain GCs is no less than 14 mm.

### scRNA-seq dataset generated from hGC

For one of the GC samples (control 1, C1), the 10xGenomics Chromium Single Cell 3’Solution was employed for capture, amplification and labeling of mRNA from single cells and for scRNA-seq library preparation. Another GC sample (control 2, C2) was processed on the DropSeq platform, and the single-cell RNAseq library was prepared. The cDNA libraries were then amplified, and the sequencing adapters were added for Illumina sequencing library preparation.

Sequencing of these libraries was performed on an Illumina HiSeq X10 system. Sequencing data (fastq files) were input into the CellRanger pipeline to align reads and generate gene-cell digital expression matrices.

### Unsupervised clustering, dimensional reduction, and data visualization

Most of the unsupervised clustering, dimensional reduction and data visualization in this paper was accomplished by a widely used scRNA-seq analytical suite, Seurat^41,42^. The Seurat objects were generated for each dataset with their digital expression matrices as input. PCA was performed by the Seurat RunPCA function. The tSNE coordinates were calculated using the Seurat RunTSNE function. Heatmaps were plotted using the Seurat DoHeatmap function. Violin plots were made using the Seurat VlnPlot function. Signal mapping was performed using the Seurat FeaturePlot function. The putative clusters were defined by the Seurat FindClusters function using the top 10 principal components and other default parameters.

### Signature gene sets for putative cell groups and gene set enrichment

The signature gene sets were defined using the Seurat FindMarkers function. This function is based on the negative binomial test of differential expression over cell clusters. The marker genes of a certain cluster were defined by significantly high expression over the other clusters in the sample. The *p*-value < 0.01 was used to select the signature gene set.

The designated gene set can be cross-compared to another gene set for significant overlap using a hypergeometric test. The correlation between the two gene sets can thus be uncovered. We may also go through a series of annotated gene sets to find the keywords (biological annotations) correlated to the signature gene set under investigation using this statistical test.

### Mice

Female C57BL/6 mice were housed in the animal laboratory with four mice per cage. All mice were kept under a 12-hour light–dark cycle at a temperature of 21 °C ± 2 °C and with a humidity of 65% ± 5%. Food and water were provided ad libitum. All experiments were performed in accordance with the National Institutes of Health Guide for the Care and Use of Laboratory Animals.

### Superovulation and sample collection

Eight-week-old female mice were injected intraperitoneally with 5 IU/mouse pregnant mare serum gonadotropin (PMSG) (Sigma–Aldrich, St. Louis, MO, USA) to stimulate follicular growth, followed 48 h later by 5 IU/mouse human chorionic gonadotropin (hCG) (Sigma–Aldrich) to induce ovulation. At different time intervals (0, 3, 6, and 9 h) after hCG administration, mice were humanely killed and their ovaries were collected. Then, mouse COCs were collected from mouse antral follicles, and cumulus GCs were isolated by the removal of all oocytes.

### Cell line cultures

The human ovarian granulosa cell lines KGN and COV434 were cultured in DMEM (Thermo Fisher Scientific, Waltham, MA, USA) supplemented with 10% fetal bovine serum (Gibco, USA) and antibiotics (100 U/ml penicillin, 100 mg/ml streptomycin, and 0.25 mg/L amphotericin B) in a humidified atmosphere of 5% CO2 and 95% air at 37 °C.

### Lentivirus production and gene transduction

Short hairpin (shRNA)-mediated knockdown of CD24 was performed using shRNA lentiviral particles (OBIO Technology, Shanghai, China) designed to suppress the expression of human CD24 in human primary GCs or COV434 cells. The cultures that were transfected with shRNA lentiviral particles containing noneffective scramble shRNA sequences (Negative control, NC) were used as the control group. The lentivirus vector constructs used for CD24 gene knockdown are as follows: pLV-mU6-[sh-scramble] EF1a-GFP-puromycin (negative control, NC, 5′-TTCTCCGAACGTGTCACGT-3′), pLV-mU6-[sh-CD24] EF1a-GFP- puromycin (CD24 shRNA_1, 5′-GGAACTTCAAGTAACTCCTCC-3′; CD24 shRNA_2, 5′-GCCAAGAAACGTCTTCTAAAT-3′). Cell infection was conducted following the protocol provided by OBIO Technology (Shanghai, China). After infection, the stable transfectants were selected using 2 mg/mL puromycin in the presence of 10% FBS for 72 h. The efficiency of CD24 downregulation in COV434 cells was confirmed by real-time polymerase chain reaction (real-time PCR) and western blotting.

### Immunofluorescence staining

COV434 cells and KGN cells were cultured on 13-mm round glass coverslips (NEST, China). After the desired treatment, culture medium was aspirated, cells were washed three times with cold PBS, fixed for 30 min in 3% paraformaldehyde and permeabilized for an additional 30 min with a permeabilization solution (0.1% Triton X-100, 5% FCS and 2% BSA [BSA] in PBS). Cells were subsequently incubated for 1 h at room temperature in the presence of anti-CD24 antibody (1:200; Thermo Fisher Scientific) and anti-EGFR antibody (1:200; Abcam, Cambridge, USA), washed three times and incubated for 1 h with goat anti-rabbit IgG H&L (ab150077, Abcam, Cambridge, USA) and goat anti-mouse IgG H&L (ab150120, Abcam, Cambridge, USA). Coverslips were washed five times in PBS and stained with Hoechst 33342 (1 mg/ml; Sigma) for 10 min. Cell samples were analyzed using an LSM 510, Zeiss laser confocal scanning microscope (Carl Zeiss, Oberkochen, Germany).

Mouse ovaries from 8-week-old mice were fixed in 4% paraformaldehyde after cryoprotection with 30% sucrose in PBS. Samples were cut transversely into 10-μm-thick sections on a cryostat. Sections were incubated with the anti-Cd24a antibody (1:1000; Abcam, Cambridge, USA) overnight at 4 °C and then with biotinylated goat anti-rat secondary antibody (1:1000; Abcam, Cambridge, USA) for 2 h at room temperature, followed by incubation with Hoechst 33342 (1 mg/ml; Sigma) for 10 min. Finally, sections were observed using a fluorescence microscope (Carl Zeiss, Oberkochen, Germany).

### Co-immunoprecipitation

COV434 and KGN Cells were lysed in buffer composed of 50 mM HEPES, pH 7.4, 150 mM NaCl, 5 mM MgCl2, 1% Triton X-100, 1 mM PMSF and a cocktail of protease inhibitors at 4 °C for 10 min and cleared by centrifugation at 12,000 rpm for 15 min at 4 °C. Protein concentrations were determined using a BCA Protein Assay Kit (P0012, Beyotime, Shanghai, China). Aliquots of cleared supernatants containing 500 µg of protein were incubated for 2 h at 4 °C with 20 µl of anti-CD24 antibodies conjugated to agarose beads at a constant rotation. The mixture was cleared by centrifugation. The supernatant after the first centrifugation was used to estimate the immunoprecipitation efficacy. The pellet was washed five times with lysis buffer devoid of Triton X-100. Proteins were removed from the beads by an appropriate sample buffer, boiled for 10 min, resolved by 10% SDS-PAGE under reducing conditions and transferred to nitrocellulose paper (Bio-Rad, Hercules, USA) for western blotting with anti-EGFR antibodies.

### Real-time polymerase chain reaction (Real-time PCR)

Total RNA from GCs was extracted with TRIzol (TAKARA, Beijing, China) and reverse-transcribed into cDNA with the High-Capacity RNA-to-cDNA Kit (TAKARA, Beijing, China) and random hexamer primers. Real-time PCR was performed using SYBR Premix Ex Taq II (TAKARA, Beijing, China) to measure duplex DNA formation with the ABI Stepone Plus system. The results were normalized to GAPDH levels. The sequences of the primer sets used are listed in Supplemental Table 6.

### Western blotting

Total cellular proteins were extracted from cells using ice-cold radioimmunoprecipitation assay (RIPA) lysis buffer (Beyotime, Shanghai, China) containing Protease/Phosphatase Inhibitor Cocktail (Cell Signaling Technology, Danvers, USA). The abundance of total protein was determined using a BCA Protein Assay Kit (P0012, Beyotime, Shanghai, China). Twenty micrograms of protein from each sample was electrophoresed on 10% SDS–polyacrylamide gels and transferred to nitrocellulose membranes (Bio-Rad, Hercules, USA). After blocking with 5% nonfat milk, the membranes were incubated with CD24 antibody (Abcam, Cambridge, USA), ERK1/2 antibody (Cell Signaling Technology, Massachusetts, USA), p-ERK1/2 antibody (Cell Signaling Technology, Danvers, USA), EGFR antibody (Abcam, Cambridge, USA), p-EGFR (Abcam, Cambridge, USA) or GAPDH (Sigma–Aldrich, Germany) overnight at 4 °C. The blots were incubated with IRdye 800-conjugated goat anti-rabbit IgG and IRdye 700-conjugated goat anti-mouse IgG and detected using an Odyssey infrared scanner (Li-COR Biosciences, Nebraska, USA). GAPDH was used as a loading control for western blotting.

### Statistical analysis

All statistical analyses in this study were performed with SPSS 18.0 software (IBM SPSS, Chicago, USA). The Mann-Whitney test was used to compare continuous variables. A *p*-value < 0.05 was considered significant.

## Supplementary information

Supplementary information

Supplementary Figure S1

Supplementary Figure S2

Supplementary Table S1 Gene set 1 list of C1 and C2

Supplementary Table S2. Gene set 2 list of C1 and C2

Supplementary Table S3. Gene set 3 list of C1 and C2

Supplementary Table S4 Results of pathway analysis using REACTOME

MATERIALSupplementary Table S5. Clinical and biochemical profiles of woman with PCOS and control

Supplementary Table S6. Primer sequences used for real-time PCR analyses

## References

[1] Baerwald AR, Adams GP, Pierson RA (2012). Ovarian antral folliculogenesis during the human menstrual cycle: a review. Hum. Reprod. Update.

[2] Hsueh AJ, Kawamura K, Cheng Y, Fauser BC (2015). Intraovarian control of early folliculogenesis. Endocr. Rev..

[3] Swain JE, Pool TB (2008). ART failure: oocyte contributions to unsuccessful fertilization. Hum. Reprod. Update.

[4] Richani D, Gilchrist RB (2018). The epidermal growth factor network: role in oocyte growth, maturation and developmental competence. Hum. Reprod. Update.

[5] Li R, Albertini DF (2013). The road to maturation: somatic cell interaction and self-organization of the mammalian oocyte. Nat. Rev. Mol. cell Biol..

[6] Persani L, Rossetti R, Di Pasquale E, Cacciatore C, Fabre S (2014). The fundamental role of bone morphogenetic protein 15 in ovarian function and its involvement in female fertility disorders. Hum. Reprod. Update.

[7] Zhang XY, Chang HM, Taylor EL, Liu RZ, Leung PCK (2018). BMP6 downregulates GDNF expression through SMAD1/5 and ERK1/2 signaling pathways in human granulosa-lutein cells. Endocrinology.

[8] Dong J (1996). Growth differentiation factor-9 is required during early ovarian folliculogenesis. Nature.

[9] Yerushalmi GM (2016). The prostaglandin transporter (PGT) as a potential mediator of ovulation. Sci. Transl. Med..

[10] Bencomo E (2006). Apoptosis of cultured granulosa-lutein cells is reduced by insulin-like growth factor I and may correlate with embryo fragmentation and pregnancy rate. Fertil. Steril..

[11] Corn CM, Hauser-Kronberger C, Moser M, Tews G, Ebner T (2005). Predictive value of cumulus cell apoptosis with regard to blastocyst development of corresponding gametes. Fertil. Steril..

[12] Gebhardt KM, Feil DK, Dunning KR, Lane M, Russell DL (2011). Human cumulus cell gene expression as a biomarker of pregnancy outcome after single embryo transfer. Fertil. Steril..

[13] McKenzie LJ (2004). Human cumulus granulosa cell gene expression: a predictor of fertilization and embryo selection in women undergoing IVF. Hum. Reprod..

[14] Assou S (2008). A non-invasive test for assessing embryo potential by gene expression profiles of human cumulus cells: a proof of concept study. Mol. Hum. Reprod..

[15] Hammond ER, Stewart B, Peek JC, Shelling AN, Cree LM (2015). Assessing embryo quality by combining non-invasive markers: early time-lapse parameters reflect gene expression in associated cumulus cells. Hum. Reprod..

[16] Dumesic DA, Abbott DH (2008). Implications of polycystic ovary syndrome on oocyte development. Semin. Reprod. Med..

[17] Dewailly D (2016). Interactions between androgens, FSH, anti-Mullerian hormone and estradiol during folliculogenesis in the human normal and polycystic ovary. Hum. Reprod. update.

[18] Pan JX (2018). Aberrant expression and DNA methylation of lipid metabolism genes in PCOS: a new insight into its pathogenesis. Clin. Epigenetics.

[19] Nilsson E (2018). Environmental toxicant induced epigenetic transgenerational inheritance of ovarian pathology and granulosa cell epigenome and transcriptome alterations: ancestral origins of polycystic ovarian syndrome and primary ovarian insufiency. Epigenetics.

[20] Kim C (2018). Chemoresistance evolution in triple-negative breast cancer delineated by single-cell sequencing. Cell.

[21] Zheng C (2017). Landscape of infiltrating T cells in liver cancer revealed by single-cell sequencing. Cell.

[22] Sidiropoulos K (2017). Reactome enhanced pathway visualization. Bioinformatics.

[23] Wissing ML (2014). Identification of new ovulation-related genes in humans by comparing the transcriptome of granulosa cells before and after ovulation triggering in the same controlled ovarian stimulation cycle. Hum. Reprod..

[24] Best SA (2014). Dual roles for Id4 in the regulation of estrogen signaling in the mammary gland and ovary. Development.

[25] Lussier JG, Diouf MN, Levesque V, Sirois J, Ndiaye K (2017). Gene expression profiling of upregulated mRNAs in granulosa cells of bovine ovulatory follicles following stimulation with hCG. Reprod. Biol. Endocrinol..

[26] Maman E (2011). Expression and regulation of sFRP family members in human granulosa cells. Mol. Hum. Reprod..

[27] Cui L (2018). GDNF-induced downregulation of miR-145-5p enhances human oocyte maturation and cumulus cell viability. J. Clin. Endocrinol. Metab..

[28] Cai G (2017). MicroRNA-145 negatively regulates cell proliferation through targeting IRS1 in isolated ovarian granulosa cells from patients with polycystic ovary syndrome. Reprod. Sci..

[29] LaVoie HA, Kordus RJ, Nguyen JB, Barth JL, Hui YY (2010). GATA depletion impacts insulin-like growth factor 1 mRNA and protein levels in luteinizing porcine granulosa cells. Biol. Reprod..

[30] Choi Y (2017). Coordinated regulation among progesterone, prostaglandins, and EGF-like factors in human ovulatory follicles. J. Clin. Endocrinol. Metab..

[31] Wang L (2015). Intracellular CD24 disrupts the ARF-NPM interaction and enables mutational and viral oncogene-mediated p53 inactivation. Nat. Commun..

[32] Lee TK (2011). CD24(+) liver tumor-initiating cells drive self-renewal and tumor initiation through STAT3-mediated NANOG regulation. Cell Stem Cell.

[33] Chan SH (2019). Identification of the novel role of CD24 as an Oncogenesis Regulator and Therapeutic Target for triple-negative breast cancer. Mol. Cancer Ther..

[34] Deng W (2016). CD24 associates with EGFR and supports EGF/EGFR signaling via RhoA in gastric cancer cells. J. Transl. Med.

[35] Tan Y, Zhao M, Xiang B, Chang C, Lu Q (2016). CD24: from a hematopoietic differentiation antigen to a genetic risk factor for multiple autoimmune diseases. Clin. Rev. Allergy Immunol..

[36] Zheng Y (2013). A rare population of CD24(+)ITGB4(+)Notch(hi) cells drives tumor propagation in NSCLC and requires Notch3 for self-renewal. Cancer Cell.

[37] Okabe H (2018). Downregulation of CD24 suppresses bone metastasis of lung cancer. Cancer Sci..

[38] Wang X (2016). CD24 promoted cancer cell angiogenesis via Hsp90-mediated STAT3/VEGF signaling pathway in colorectal cancer. Oncotarget.

[39] Rotterdam EA-SPCWG (2004). Revised 2003 consensus on diagnostic criteria and long-term health risks related to polycystic ovary syndrome (PCOS). Hum. Reprod..

[40] Tarlatzis BC (2006). GnRH antagonists in ovarian stimulation for IVF. Hum. Reprod. update.

[41] Butler A, Hoffman P, Smibert P, Papalexi E, Satija R (2018). Integrating single-cell transcriptomic data across different conditions, technologies, and species. Nat. Biotechnol..

[42] Macosko EZ (2015). Highly parallel genome-wide expression profiling of individual cells using nanoliter droplets. Cell.

