# Supplementary material for: CD24: a marker of granulosa cell subpopulation and a mediator of ovulation
Source: Cell Death Dis. 2019 Oct 17;10(11):791. doi: 10.1038/s41419-019-1995-1 (PMC6797718; doi:10.1038/s41419-019-1995-1)
Supplement: Supplementary file 1 — Supplementary information [file 41419_2019_1995_MOESM1_ESM.docx]

**Supplementary figure legends**

**Supplementary Figure 1. Expression levels of known key genes of cumulus GCs.** (A) Heatmap of the mean expression of known key genes within each subpopulation in C2. Cells are classified into three clusters (bottom bars). (B) t-SNE plots for expression of CD24 in C2. Each yellow point represents a single cell. The color gradient in the t-SNE plot represents the relative expression level of CD24 in a cell across the whole population and subpopulations: (light red) low; (dark red) high. (C) Violin plots for expression of CD24, [prostaglandin](javascript:;) synthases (ARK1C1, PTGS2, PTGES and PLA2G4A) and [prostaglandin](javascript:;) transporters (SLCO2A1 and ABCC4) in C2. Each point represents a single cell.

**Supplementary Figure 2. ERK1/2 pathway activation supports the expression of** [**prostaglandin**](javascript:;) **metabolism-related genes.** FSH-pretreated human cultured GCs (48 hours) were initially pretreated with or without U0126 [an inhibitor of MEK (mitogen-activated protein kinase kinase), an upstream activator of ERK; 10 mM] for 1 hour and then reincubated with hCG (1 U/ml) for 24 hours. The expression levels of [prostaglandin](javascript:;) synthases (ARK1C1, PTGS2, PTGES and PLA2G4A) and [prostaglandin](javascript:;) transporters (SLCO2A1 and ABCC4) were detected using real-time PCR and normalized against GAPDH. *p<0.05.
