# Supplementary figures and images for: CD24: a marker of granulosa cell subpopulation and a mediator of ovulation
Source: Cell Death Dis. 2019 Oct 17;10(11):791. doi: 10.1038/s41419-019-1995-1 (PMC6797718; doi:10.1038/s41419-019-1995-1)

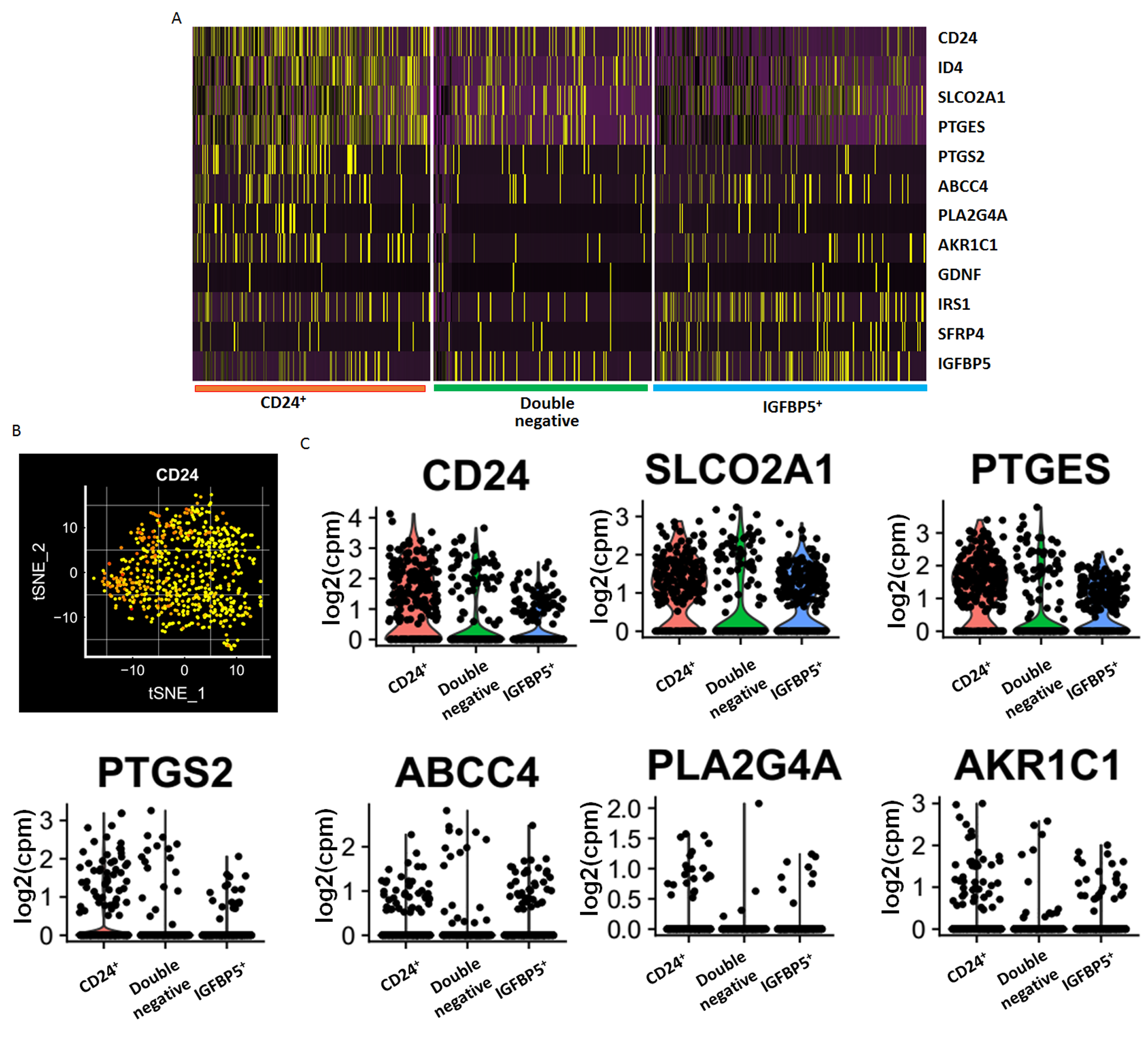

Supplement: Supplementary file 2 — Supplementary Figure S1 [file 41419_2019_1995_MOESM2_ESM.tif]

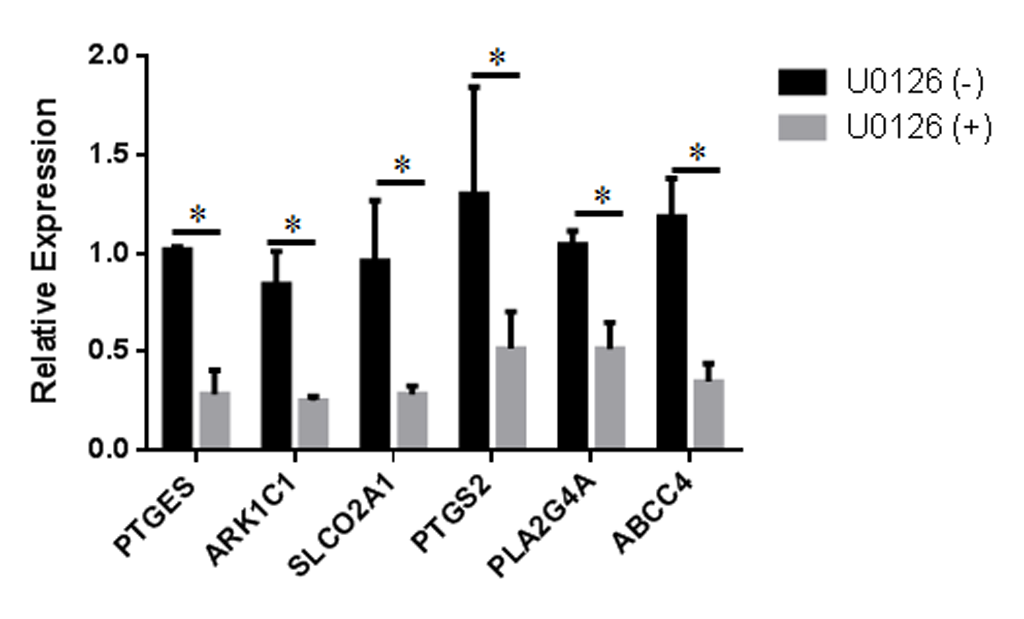

Supplement: Supplementary file 3 — Supplementary Figure S2 [file 41419_2019_1995_MOESM3_ESM.tif]
