# Supplementary material for: CD24: a marker of granulosa cell subpopulation and a mediator of ovulation
Source: Cell Death Dis. 2019 Oct 17;10(11):791. doi: 10.1038/s41419-019-1995-1 (PMC6797718; doi:10.1038/s41419-019-1995-1)
Supplement: Supplementary file 4 — Supplementary Table S1 Gene set 1 list of C1 and C2 [file 41419_2019_1995_MOESM4_ESM.pdf]

Control 1 (C1)

p_value is from the negantive-binomial test for the detection differential genes over the two cell groups.
avg_logFC is for the average logorithm values of fold difference.
pct.1 is for the percentage of cells that gives the signal on the designate gene on the target cell group/cluster.
pct.2 is for the percentage of cells that give the signal on the designate gene on the counterpart of the target cell group/cluster.

gene name	p_val	avg_logFC	pct.1	pct.2	p_val_adj	Cluster_Ids
UGP2	1.04E-34	0.950975685	0.908	0.568	3.50E-30	CD24+
CPM	1.43E-29	0.676703619	0.95	0.668	4.80E-25	CD24+
SAT1	1.06E-28	0.55152349	0.986	0.898	3.58E-24	CD24+
MT2A	4.34E-27	0.745834952	0.991	0.927	1.46E-22	CD24+
CD24	5.24E-24	0.825322115	0.683	0.285	1.77E-19	CD24+
SEC14L2	1.87E-22	0.448208106	0.564	0.192	6.31E-18	CD24+
SRGN	1.88E-22	0.81960172	0.564	0.212	6.32E-18	CD24+
SMARCA1	8.60E-22	0.636089377	0.968	0.791	2.89E-17	CD24+
PTGES	1.82E-21	0.63049056	0.794	0.441	6.14E-17	CD24+
HSD11B1	2.87E-21	0.6440465	0.913	0.644	9.65E-17	CD24+
RGS2	5.30E-20	0.576207364	0.376	0.091	1.78E-15	CD24+
PRKX	1.80E-19	0.436166988	0.995	0.793	6.06E-15	CD24+
PEMT	1.39E-17	0.628694792	0.936	0.706	4.69E-13	CD24+
SMC6	1.78E-17	0.707089324	0.661	0.343	5.98E-13	CD24+
PDLIM1	2.94E-17	0.457433658	0.674	0.321	9.91E-13	CD24+
KIF13B	3.45E-16	0.434545224	0.867	0.552	1.16E-11	CD24+
GTF3C6	1.03E-15	0.442966255	0.716	0.381	3.47E-11	CD24+
ID4	3.09E-15	0.589318578	0.697	0.388	1.04E-10	CD24+
DBI	3.79E-15	0.48977489	0.89	0.65	1.28E-10	CD24+
SFRP5	1.06E-14	0.465239049	0.44	0.169	3.58E-10	CD24+
CRISPLD2	2.64E-14	0.353809444	0.633	0.321	8.89E-10	CD24+
FAM83A-AS1	3.06E-14	0.508796874	0.514	0.216	1.03E-09	CD24+
ATAD2	5.23E-14	0.424984475	0.532	0.234	1.76E-09	CD24+
RAD51C	6.08E-14	0.383086491	0.596	0.287	2.05E-09	CD24+
ADAMTS9	7.04E-14	0.46853662	0.372	0.125	2.37E-09	CD24+
VMP1	1.14E-13	0.361655866	0.826	0.503	3.83E-09	CD24+
SLC6A6	1.43E-13	0.37868069	0.628	0.314	4.82E-09	CD24+
SAMHD1	1.67E-13	0.425696333	0.734	0.448	5.62E-09	CD24+

ALAD	1.70E-13	0.462895578	0.528	0.241	5.72E-09	CD24+
RAMP1	1.90E-13	0.442024318	0.413	0.149	6.38E-09	CD24+
ASH1L	3.21E-13	0.28458201	0.61	0.29	1.08E-08	CD24+
EMP2	3.66E-13	0.346986607	0.573	0.274	1.23E-08	CD24+
CST2	3.80E-13	0.625129926	0.427	0.171	1.28E-08	CD24+
MYO1B	4.07E-13	0.411287329	0.477	0.207	1.37E-08	CD24+
ARHGAP18	6.33E-13	0.335917664	0.463	0.189	2.13E-08	CD24+
SSX2IP	7.71E-13	0.384119652	0.317	0.098	2.59E-08	CD24+
FKBP11	9.20E-13	0.412364156	0.578	0.298	3.10E-08	CD24+
RASD1	1.12E-12	0.465625408	0.427	0.176	3.77E-08	CD24+
IGFBP2	1.29E-12	0.341799719	0.729	0.428	4.33E-08	CD24+
TECRL	1.64E-12	0.509336429	0.349	0.122	5.52E-08	CD24+
PDIA5	1.72E-12	0.311190963	0.408	0.158	5.80E-08	CD24+
ZFR	1.77E-12	0.450737257	0.734	0.443	5.97E-08	CD24+
MDH1	1.81E-12	0.398333688	0.752	0.45	6.09E-08	CD24+
TMEM132C	3.11E-12	0.297200708	0.427	0.169	1.05E-07	CD24+
TNFAIP3	8.21E-12	0.335802232	0.454	0.2	2.76E-07	CD24+
PHACTR4	8.25E-12	0.420835136	0.78	0.494	2.78E-07	CD24+
LAMB1	1.02E-11	0.360903081	0.486	0.229	3.43E-07	CD24+
FAM104A	1.16E-11	0.421067512	0.381	0.154	3.92E-07	CD24+
IRS2	1.24E-11	0.365024886	0.894	0.621	4.16E-07	CD24+
AMOTL1	2.44E-11	0.315336925	0.743	0.43	8.21E-07	CD24+
GATA6-AS1	2.74E-11	0.361439292	0.431	0.192	9.24E-07	CD24+
GADD45A	3.57E-11	0.387149034	0.849	0.572	1.20E-06	CD24+
CBFB	6.39E-11	0.25185358	0.592	0.307	2.15E-06	CD24+
TIPARP	7.83E-11	0.40624464	0.312	0.111	2.64E-06	CD24+
STRBP	1.63E-10	0.317458614	0.514	0.272	5.50E-06	CD24+
SYAP1	1.65E-10	0.309434311	0.693	0.396	5.56E-06	CD24+
NR2F2	1.75E-10	0.301999171	0.491	0.236	5.89E-06	CD24+
GAL	1.81E-10	0.857098638	0.165	0.029	6.10E-06	CD24+
TGFBR3	1.98E-10	0.42327914	0.339	0.134	6.65E-06	CD24+
TAF1D	2.06E-10	0.345155064	0.743	0.479	6.94E-06	CD24+
GNG5	2.09E-10	0.355084931	0.849	0.599	7.03E-06	CD24+
C1R	2.97E-10	0.313632672	0.697	0.41	9.99E-06	CD24+
NUDT16	3.24E-10	0.324113587	0.683	0.414	1.09E-05	CD24+
SLCO2A1	3.40E-10	0.306728499	0.757	0.461	1.15E-05	CD24+

RPS7	4.30E-10	0.292364843	0.913	0.666	1.45E-05	CD24+
ABHD12	4.80E-10	0.289347621	0.642	0.385	1.61E-05	CD24+
SKIL	4.98E-10	0.31955233	0.39	0.171	1.68E-05	CD24+
RBM22	5.35E-10	0.50944579	0.573	0.332	1.80E-05	CD24+
TMEM120A	6.82E-10	0.263137448	0.505	0.256	2.30E-05	CD24+
CUL4B	6.84E-10	0.376918199	0.569	0.316	2.30E-05	CD24+
ELL2	8.12E-10	0.266128089	0.468	0.229	2.73E-05	CD24+
APPL2	9.26E-10	0.311241674	0.651	0.394	3.12E-05	CD24+
PTGS2	9.36E-10	0.514430845	0.252	0.08	3.15E-05	CD24+
WDR60	9.93E-10	0.374016269	0.578	0.323	3.34E-05	CD24+
ARHGAP6	1.33E-09	0.317640272	0.803	0.521	4.48E-05	CD24+
PLEKHH2	1.39E-09	0.268513088	0.362	0.156	4.69E-05	CD24+
ZNF189	1.51E-09	0.268318393	0.358	0.151	5.08E-05	CD24+
KIAA1324L	1.68E-09	0.348442523	0.275	0.098	5.64E-05	CD24+
FST	1.76E-09	0.430340231	0.505	0.263	5.92E-05	CD24+
KLF6	1.82E-09	0.290756858	0.972	0.84	6.12E-05	CD24+
DNAJC1	1.91E-09	0.28760082	0.592	0.323	6.42E-05	CD24+
TOP1	2.46E-09	0.324706705	0.761	0.49	8.29E-05	CD24+
ZFP36	2.49E-09	0.276616239	0.573	0.323	8.38E-05	CD24+
CLU	2.70E-09	0.350654503	0.931	0.748	9.09E-05	CD24+
FAM46A	2.72E-09	0.31039204	0.514	0.263	9.15E-05	CD24+
PKP2	3.86E-09	0.295779758	0.358	0.158	0.000129957	CD24+
CEP89	4.00E-09	0.262074208	0.45	0.22	0.000134531	CD24+
C5orf30	4.18E-09	0.277721979	0.289	0.109	0.000140734	CD24+
GPC4	4.85E-09	0.333871998	0.266	0.096	0.000163357	CD24+
SPON2	5.82E-09	0.492416801	0.674	0.423	0.000195776	CD24+
7-Sep	6.62E-09	0.294512653	0.817	0.568	0.000222744	CD24+
WDR44	8.14E-09	0.317075316	0.307	0.129	0.000273865	CD24+
CD63	8.84E-09	0.279982912	0.972	0.842	0.000297482	CD24+
GBA2	9.87E-09	0.258534946	0.482	0.252	0.000332163	CD24+
ARGLU1	1.30E-08	0.356649887	0.757	0.514	0.000438631	CD24+
SRPX2	1.89E-08	0.264667354	0.587	0.343	0.000636288	CD24+
KIAA2026	2.11E-08	0.270855939	0.55	0.325	0.000708979	CD24+
TMEM37	2.29E-08	0.362570766	0.454	0.241	0.00077233	CD24+
FBXO25	2.52E-08	0.256497057	0.39	0.192	0.00084957	CD24+
ACTN2	2.84E-08	0.290024395	0.431	0.232	0.000956162	CD24+

UPF3A	3.11E-08	0.292165452	0.459	0.243	0.001048344	CD24+
HSD17B1	3.19E-08	0.254147537	0.362	0.169	0.001074627	CD24+
FKBP5	3.44E-08	0.252774414	0.954	0.824	0.001157325	CD24+
EIF4ENIF1	3.75E-08	0.303928491	0.294	0.122	0.00126147	CD24+
S100B	4.01E-08	0.28863625	0.193	0.058	0.001350746	CD24+
CMAS	4.35E-08	0.304218513	0.821	0.597	0.001464276	CD24+
ADAMTS1	4.76E-08	0.359292578	0.427	0.225	0.00160236	CD24+
HILPDA	5.06E-08	0.409611299	0.335	0.151	0.00170271	CD24+
MMD	8.77E-08	0.361171158	0.353	0.174	0.002952311	CD24+
RHOU	8.89E-08	0.317691173	0.624	0.381	0.002991827	CD24+
NR4A1	9.28E-08	0.277790205	0.716	0.479	0.00312313	CD24+
HS3ST1	1.98E-07	0.386814519	0.22	0.082	0.006677839	CD24+
EREG	2.08E-07	0.366087296	0.312	0.143	0.006995504	CD24+
IDI1	2.56E-07	0.253088807	0.56	0.33	0.008602057	CD24+
DUSP6	2.87E-07	0.268470532	0.266	0.111	0.009673048	CD24+

Control 2 (C2)

p_value is from the negantive-binomial test for the detection differential genes over the two cell groups.
avg_logFC is for the average logorithm values of fold difference.
pct.1 is for the percentage of cells that gives the signal on the designate gene on the target cell group/cluster.
pct.2 is for the percentage of cells that give the signal on the designate gene on the counterpart of the target cell group/cluster.

gene name	p_val	avg_logFC	pct.1	pct.2	p_val_adj	Cluster_Ids
UGP2	1.04E-34	0.950975685	0.908	0.568	3.50E-30	CD24+
CPM	1.43E-29	0.676703619	0.95	0.668	4.80E-25	CD24+
SAT1	1.06E-28	0.55152349	0.986	0.898	3.58E-24	CD24+
MT2A	4.34E-27	0.745834952	0.991	0.927	1.46E-22	CD24+
CD24	5.24E-24	0.825322115	0.683	0.285	1.77E-19	CD24+
SEC14L2	1.87E-22	0.448208106	0.564	0.192	6.31E-18	CD24+
SRGN	1.88E-22	0.81960172	0.564	0.212	6.32E-18	CD24+
SMARCA1	8.60E-22	0.636089377	0.968	0.791	2.89E-17	CD24+
PTGES	1.82E-21	0.63049056	0.794	0.441	6.14E-17	CD24+
HSD11B1	2.87E-21	0.6440465	0.913	0.644	9.65E-17	CD24+
RGS2	5.30E-20	0.576207364	0.376	0.091	1.78E-15	CD24+
PRKX	1.80E-19	0.436166988	0.995	0.793	6.06E-15	CD24+
PEMT	1.39E-17	0.628694792	0.936	0.706	4.69E-13	CD24+
SMC6	1.78E-17	0.707089324	0.661	0.343	5.98E-13	CD24+
PDLIM1	2.94E-17	0.457433658	0.674	0.321	9.91E-13	CD24+
KIF13B	3.45E-16	0.434545224	0.867	0.552	1.16E-11	CD24+
GTF3C6	1.03E-15	0.442966255	0.716	0.381	3.47E-11	CD24+
ID4	3.09E-15	0.589318578	0.697	0.388	1.04E-10	CD24+
DBI	3.79E-15	0.48977489	0.89	0.65	1.28E-10	CD24+
SFRP5	1.06E-14	0.465239049	0.44	0.169	3.58E-10	CD24+
CRISPLD2	2.64E-14	0.353809444	0.633	0.321	8.89E-10	CD24+
FAM83A-AS1	3.06E-14	0.508796874	0.514	0.216	1.03E-09	CD24+
ATAD2	5.23E-14	0.424984475	0.532	0.234	1.76E-09	CD24+
RAD51C	6.08E-14	0.383086491	0.596	0.287	2.05E-09	CD24+
ADAMTS9	7.04E-14	0.46853662	0.372	0.125	2.37E-09	CD24+
VMP1	1.14E-13	0.361655866	0.826	0.503	3.83E-09	CD24+
SLC6A6	1.43E-13	0.37868069	0.628	0.314	4.82E-09	CD24+
SAMHD1	1.67E-13	0.425696333	0.734	0.448	5.62E-09	CD24+

ALAD	1.70E-13	0.462895578	0.528	0.241	5.72E-09	CD24+
RAMP1	1.90E-13	0.442024318	0.413	0.149	6.38E-09	CD24+
ASH1L	3.21E-13	0.28458201	0.61	0.29	1.08E-08	CD24+
EMP2	3.66E-13	0.346986607	0.573	0.274	1.23E-08	CD24+
CST2	3.80E-13	0.625129926	0.427	0.171	1.28E-08	CD24+
MYO1B	4.07E-13	0.411287329	0.477	0.207	1.37E-08	CD24+
ARHGAP18	6.33E-13	0.335917664	0.463	0.189	2.13E-08	CD24+
SSX2IP	7.71E-13	0.384119652	0.317	0.098	2.59E-08	CD24+
FKBP11	9.20E-13	0.412364156	0.578	0.298	3.10E-08	CD24+
RASD1	1.12E-12	0.465625408	0.427	0.176	3.77E-08	CD24+
IGFBP2	1.29E-12	0.341799719	0.729	0.428	4.33E-08	CD24+
TECRL	1.64E-12	0.509336429	0.349	0.122	5.52E-08	CD24+
PDIA5	1.72E-12	0.311190963	0.408	0.158	5.80E-08	CD24+
ZFR	1.77E-12	0.450737257	0.734	0.443	5.97E-08	CD24+
MDH1	1.81E-12	0.398333688	0.752	0.45	6.09E-08	CD24+
TMEM132C	3.11E-12	0.297200708	0.427	0.169	1.05E-07	CD24+
TNFAIP3	8.21E-12	0.335802232	0.454	0.2	2.76E-07	CD24+
PHACTR4	8.25E-12	0.420835136	0.78	0.494	2.78E-07	CD24+
LAMB1	1.02E-11	0.360903081	0.486	0.229	3.43E-07	CD24+
FAM104A	1.16E-11	0.421067512	0.381	0.154	3.92E-07	CD24+
IRS2	1.24E-11	0.365024886	0.894	0.621	4.16E-07	CD24+
AMOTL1	2.44E-11	0.315336925	0.743	0.43	8.21E-07	CD24+
GATA6-AS1	2.74E-11	0.361439292	0.431	0.192	9.24E-07	CD24+
GADD45A	3.57E-11	0.387149034	0.849	0.572	1.20E-06	CD24+
CBFB	6.39E-11	0.25185358	0.592	0.307	2.15E-06	CD24+
TIPARP	7.83E-11	0.40624464	0.312	0.111	2.64E-06	CD24+
STRBP	1.63E-10	0.317458614	0.514	0.272	5.50E-06	CD24+
SYAP1	1.65E-10	0.309434311	0.693	0.396	5.56E-06	CD24+
NR2F2	1.75E-10	0.301999171	0.491	0.236	5.89E-06	CD24+
GAL	1.81E-10	0.857098638	0.165	0.029	6.10E-06	CD24+
TGFBR3	1.98E-10	0.42327914	0.339	0.134	6.65E-06	CD24+
TAF1D	2.06E-10	0.345155064	0.743	0.479	6.94E-06	CD24+
GNG5	2.09E-10	0.355084931	0.849	0.599	7.03E-06	CD24+
C1R	2.97E-10	0.313632672	0.697	0.41	9.99E-06	CD24+
NUDT16	3.24E-10	0.324113587	0.683	0.414	1.09E-05	CD24+
SLCO2A1	3.40E-10	0.306728499	0.757	0.461	1.15E-05	CD24+

RPS7	4.30E-10	0.292364843	0.913	0.666	1.45E-05	CD24+
ABHD12	4.80E-10	0.289347621	0.642	0.385	1.61E-05	CD24+
SKIL	4.98E-10	0.31955233	0.39	0.171	1.68E-05	CD24+
RBM22	5.35E-10	0.50944579	0.573	0.332	1.80E-05	CD24+
TMEM120A	6.82E-10	0.263137448	0.505	0.256	2.30E-05	CD24+
CUL4B	6.84E-10	0.376918199	0.569	0.316	2.30E-05	CD24+
ELL2	8.12E-10	0.266128089	0.468	0.229	2.73E-05	CD24+
APPL2	9.26E-10	0.311241674	0.651	0.394	3.12E-05	CD24+
PTGS2	9.36E-10	0.514430845	0.252	0.08	3.15E-05	CD24+
WDR60	9.93E-10	0.374016269	0.578	0.323	3.34E-05	CD24+
ARHGAP6	1.33E-09	0.317640272	0.803	0.521	4.48E-05	CD24+
PLEKHH2	1.39E-09	0.268513088	0.362	0.156	4.69E-05	CD24+
ZNF189	1.51E-09	0.268318393	0.358	0.151	5.08E-05	CD24+
KIAA1324L	1.68E-09	0.348442523	0.275	0.098	5.64E-05	CD24+
FST	1.76E-09	0.430340231	0.505	0.263	5.92E-05	CD24+
KLF6	1.82E-09	0.290756858	0.972	0.84	6.12E-05	CD24+
DNAJC1	1.91E-09	0.28760082	0.592	0.323	6.42E-05	CD24+
TOP1	2.46E-09	0.324706705	0.761	0.49	8.29E-05	CD24+
ZFP36	2.49E-09	0.276616239	0.573	0.323	8.38E-05	CD24+
CLU	2.70E-09	0.350654503	0.931	0.748	9.09E-05	CD24+
FAM46A	2.72E-09	0.31039204	0.514	0.263	9.15E-05	CD24+
PKP2	3.86E-09	0.295779758	0.358	0.158	0.000129957	CD24+
CEP89	4.00E-09	0.262074208	0.45	0.22	0.000134531	CD24+
C5orf30	4.18E-09	0.277721979	0.289	0.109	0.000140734	CD24+
GPC4	4.85E-09	0.333871998	0.266	0.096	0.000163357	CD24+
SPON2	5.82E-09	0.492416801	0.674	0.423	0.000195776	CD24+
7-Sep	6.62E-09	0.294512653	0.817	0.568	0.000222744	CD24+
WDR44	8.14E-09	0.317075316	0.307	0.129	0.000273865	CD24+
CD63	8.84E-09	0.279982912	0.972	0.842	0.000297482	CD24+
GBA2	9.87E-09	0.258534946	0.482	0.252	0.000332163	CD24+
ARGLU1	1.30E-08	0.356649887	0.757	0.514	0.000438631	CD24+
SRPX2	1.89E-08	0.264667354	0.587	0.343	0.000636288	CD24+
KIAA2026	2.11E-08	0.270855939	0.55	0.325	0.000708979	CD24+
TMEM37	2.29E-08	0.362570766	0.454	0.241	0.00077233	CD24+
FBXO25	2.52E-08	0.256497057	0.39	0.192	0.00084957	CD24+
ACTN2	2.84E-08	0.290024395	0.431	0.232	0.000956162	CD24+

UPF3A	3.11E-08	0.292165452	0.459	0.243	0.001048344	CD24+
HSD17B1	3.19E-08	0.254147537	0.362	0.169	0.001074627	CD24+
FKBP5	3.44E-08	0.252774414	0.954	0.824	0.001157325	CD24+
EIF4ENIF1	3.75E-08	0.303928491	0.294	0.122	0.00126147	CD24+
S100B	4.01E-08	0.28863625	0.193	0.058	0.001350746	CD24+
CMAS	4.35E-08	0.304218513	0.821	0.597	0.001464276	CD24+
ADAMTS1	4.76E-08	0.359292578	0.427	0.225	0.00160236	CD24+
HILPDA	5.06E-08	0.409611299	0.335	0.151	0.00170271	CD24+
MMD	8.77E-08	0.361171158	0.353	0.174	0.002952311	CD24+
RHOU	8.89E-08	0.317691173	0.624	0.381	0.002991827	CD24+
NR4A1	9.28E-08	0.277790205	0.716	0.479	0.00312313	CD24+
HS3ST1	1.98E-07	0.386814519	0.22	0.082	0.006677839	CD24+
EREG	2.08E-07	0.366087296	0.312	0.143	0.006995504	CD24+
IDI1	2.56E-07	0.253088807	0.56	0.33	0.008602057	CD24+
DUSP6	2.87E-07	0.268470532	0.266	0.111	0.009673048	CD24+
