# Supplementary material for: CD24: a marker of granulosa cell subpopulation and a mediator of ovulation
Source: Cell Death Dis. 2019 Oct 17;10(11):791. doi: 10.1038/s41419-019-1995-1 (PMC6797718; doi:10.1038/s41419-019-1995-1)
Supplement: Supplementary file 5 — Supplementary Table S2. Gene set 2 list of C1 and C2 [file 41419_2019_1995_MOESM5_ESM.pdf]

Control 1 (C1)

p_value is from the negantive-binomial test for the detection differential genes over the two cell groups.
avg_logFC is for the average logorithm values of fold difference.
pct.1 is for the percentage of cells that gives the signal on the designate gene on the target cell group/cluster.
pct.2 is for the percentage of cells that give the signal on the designate gene on the counterpart of the target cell group/cluster.

gene name	p_val	avg_logFC	pct.1	pct.2	p_val_adj	Cluster_Ids
GSTM3	4.79583E-27	0.519375389	1	1	1.61591E-22	Double negative
DIRAS3	5.69068E-27	0.90488752	0.975	0.912	1.91742E-22	Double negative
STAR	3.49795E-26	0.712993039	0.993	0.971	1.1786E-21	Double negative
NREP	6.11354E-26	0.526371377	0.975	0.96	2.0599E-21	Double negative
FDX1	3.00041E-25	0.737531069	0.993	0.974	1.01096E-20	Double negative
SEMA3A	6.31933E-23	0.421728374	0.998	0.942	2.12923E-18	Double negative
MRO	1.06682E-21	0.624002329	0.965	0.931	3.59456E-17	Double negative
HSD3B2	3.60161E-20	0.865778975	0.96	0.887	1.21352E-15	Double negative
AQP11	2.12188E-18	0.401383396	0.754	0.511	7.14945E-14	Double negative
LSAMP	2.25019E-17	0.330093584	0.789	0.54	7.58179E-13	Double negative
CYCS	4.94577E-17	0.333044424	0.975	0.964	1.66643E-12	Double negative
NR5A2	1.01516E-16	0.311147262	0.918	0.792	3.42048E-12	Double negative
RDX	2.82259E-16	0.38521062	1	0.993	9.51045E-12	Double negative
S100A16	8.83284E-16	0.276228931	0.995	0.996	2.97614E-11	Double negative
ATP2B4	2.04029E-15	0.366063647	0.958	0.901	6.87454E-11	Double negative
TCEAL3	2.05262E-15	0.30265261	0.97	0.934	6.9161E-11	Double negative
KLHL4	2.68595E-15	0.261548218	0.96	0.883	9.05004E-11	Double negative
SPARC	2.19052E-14	0.383712364	1	1	7.38073E-10	Double negative
AREG	3.16721E-14	0.692950534	0.983	0.949	1.06716E-09	Double negative
TM4SF1	1.31179E-13	0.423109846	0.98	0.92	4.41996E-09	Double negative
SMIM14	6.67776E-13	0.276147883	0.958	0.898	2.25E-08	Double negative
GPC6	7.8821E-13	0.277466466	0.938	0.843	2.6558E-08	Double negative
IFITM3	1.50859E-12	0.262006954	0.995	1	5.08303E-08	Double negative
ADAMTS9	3.04909E-12	0.322148788	0.659	0.453	1.02736E-07	Double negative
MT3	3.13764E-12	0.479496842	0.647	0.431	1.0572E-07	Double negative
BEX1	4.12769E-12	0.407429844	0.983	0.967	1.39079E-07	Double negative
C12orf75	5.1986E-12	0.297881558	0.878	0.774	1.75161E-07	Double negative
C7	5.87541E-12	0.408188284	0.833	0.679	1.97966E-07	Double negative

G0S2	6.88852E-12	0.523568467	0.965	0.985	2.32102E-07	Double negative
PAPPA	1.1577E-11	0.43846829	0.771	0.573	3.90075E-07	Double negative
HMGCS1	1.45398E-11	0.373105898	0.945	0.894	4.89904E-07	Double negative
ANXA5	5.4601E-11	0.257016488	1	0.996	1.83973E-06	Double negative
S100A6	5.4748E-11	0.282517908	1	1	1.84468E-06	Double negative
HSPB1	1.87945E-10	0.314889059	1	0.996	6.33263E-06	Double negative
LGALS1	2.65091E-10	0.312329033	1	0.993	8.93199E-06	Double negative
SRPX	3.61533E-10	0.391744065	1	1	1.21815E-05	Double negative
PGK1	6.90859E-10	0.254598076	0.983	0.956	2.32778E-05	Double negative
AADAC	8.90605E-10	0.267945074	0.764	0.562	3.00081E-05	Double negative
MGST1	3.47861E-09	0.319049147	0.985	0.942	0.000117208	Double negative
PPP1R14A	3.38827E-08	0.326054513	0.619	0.489	0.001141644	Double negative
ATP1A1	4.69011E-08	0.277436521	0.995	0.993	0.001580287	Double negative

Control 2 (C2)

p_value is from the negantive-binomial test for the detection differential genes over the two cell groups.
avg_logFC is for the average logorithm values of fold difference.
pct.1 is for the percentage of cells that gives the signal on the designate gene on the target cell group/cluster.
pct.2 is for the percentage of cells that give the signal on the designate gene on the counterpart of the target cell group/cluster.

gene name	p_val	avg_logFC	pct.1	pct.2	p_val_adj	Cluster_Ids
