# Supplementary material for: CD24: a marker of granulosa cell subpopulation and a mediator of ovulation
Source: Cell Death Dis. 2019 Oct 17;10(11):791. doi: 10.1038/s41419-019-1995-1 (PMC6797718; doi:10.1038/s41419-019-1995-1)
Supplement: Supplementary file 6 — Supplementary Table S3. Gene set 3 list of C1 and C2 [file 41419_2019_1995_MOESM6_ESM.pdf]

Control 1 (C1)

p_value is from the negantive-binomial test for the detection differential genes over the two cell groups.
avg_logFC is for the average logorithm values of fold difference.
pct.1 is for the percentage of cells that gives the signal on the designate gene on the target cell group/cluster.
pct.2 is for the percentage of cells that give the signal on the designate gene on the counterpart of the target cell group/cluster.

gene name	p_val	avg_logFC	pct.1	pct.2	p_val_adj	Cluster_Ids
SFRP4	3.27E-49	1.831389554	0.807	0.237	1.10E-44	IGFBP5+
PALLD	1.05E-43	1.221991742	0.982	0.73	3.53E-39	IGFBP5+
IGFBP5	4.90E-42	1.971660515	1	0.84	1.65E-37	IGFBP5+
THBS1	1.74E-38	1.302219094	1	0.92	5.86E-34	IGFBP5+
COL1A2	2.01E-38	1.216540723	0.904	0.445	6.76E-34	IGFBP5+
SMS	1.06E-37	0.775964887	1	0.961	3.58E-33	IGFBP5+
GJA1	1.07E-36	0.931776838	1	0.973	3.61E-32	IGFBP5+
IGFBP7	6.97E-31	0.757985466	1	0.996	2.35E-26	IGFBP5+
RDH10	1.08E-30	0.71440814	0.605	0.158	3.65E-26	IGFBP5+
GDNF	3.86E-30	0.32376516	0.439	0.06	1.30E-25	IGFBP5+
STMN1	5.40E-29	0.850294697	0.965	0.938	1.82E-24	IGFBP5+
CTNNAL1	2.51E-28	0.754175978	0.982	0.941	8.46E-24	IGFBP5+
ACSM3	4.13E-28	0.854967645	0.965	0.639	1.39E-23	IGFBP5+
KLHDC8A	4.44E-28	0.475171119	0.675	0.23	1.50E-23	IGFBP5+
CRIM1	1.00E-27	0.45747562	0.763	0.302	3.38E-23	IGFBP5+
FILIP1L	1.72E-27	0.824436511	0.842	0.489	5.79E-23	IGFBP5+
HTRA1	2.48E-26	0.931860941	0.982	0.954	8.35E-22	IGFBP5+
DAAM1	6.74E-26	0.698003611	0.947	0.788	2.27E-21	IGFBP5+
MARCKSL1	8.34E-26	0.64196881	0.991	0.929	2.81E-21	IGFBP5+
HMCN1	1.33E-25	0.498353078	0.658	0.226	4.48E-21	IGFBP5+
ANTXR1	2.88E-25	0.435117577	0.842	0.427	9.69E-21	IGFBP5+
F3	4.84E-25	1.269047777	0.842	0.552	1.63E-20	IGFBP5+
CCDC80	6.02E-25	0.347727153	0.491	0.11	2.03E-20	IGFBP5+
PTMA	6.43E-25	0.730231734	1	0.996	2.17E-20	IGFBP5+
FHL2	1.30E-24	0.710190616	0.982	0.957	4.36E-20	IGFBP5+
CYR61	3.78E-24	1.51116733	0.956	0.907	1.27E-19	IGFBP5+
SOX4	3.52E-23	1.268119356	0.711	0.361	1.19E-18	IGFBP5+
PHACTR2	3.57E-23	0.478006037	0.825	0.468	1.20E-18	IGFBP5+

ARL4C	4.64E-23	0.491936601	0.596	0.196	1.56E-18	IGFBP5+
RP11-277P12.20	5.24E-23	0.62307141	0.746	0.338	1.77E-18	IGFBP5+
P3H2	5.39E-23	0.590440158	1	0.922	1.82E-18	IGFBP5+
COL3A1	6.70E-23	0.557362108	0.746	0.311	2.26E-18	IGFBP5+
EBPL	6.77E-23	0.319824449	0.675	0.265	2.28E-18	IGFBP5+
B4GALT5	1.90E-22	0.588690787	0.956	0.831	6.41E-18	IGFBP5+
VIM	2.97E-22	0.630968028	1	1	1.00E-17	IGFBP5+
SPATS2	3.04E-22	0.489856164	0.939	0.799	1.03E-17	IGFBP5+
EXT1	3.11E-22	0.598364279	0.982	0.776	1.05E-17	IGFBP5+
RNASE1	3.66E-22	0.642335901	0.825	0.496	1.23E-17	IGFBP5+
JUP	3.92E-22	0.315923842	0.684	0.279	1.32E-17	IGFBP5+
CLEC4M	1.68E-21	0.294906962	0.404	0.084	5.65E-17	IGFBP5+
EPHX1	1.86E-21	0.549897331	0.912	0.705	6.28E-17	IGFBP5+
NEXN	2.02E-21	0.417996408	0.64	0.265	6.80E-17	IGFBP5+
NTN4	1.01E-20	0.385890105	0.605	0.221	3.42E-16	IGFBP5+
NAP1L1	1.29E-20	0.360423786	0.991	0.973	4.36E-16	IGFBP5+
IRS1	2.01E-20	0.512378217	0.746	0.397	6.76E-16	IGFBP5+
MDK	2.30E-20	0.653414155	0.991	0.991	7.75E-16	IGFBP5+
NRP2	5.76E-20	0.363935125	0.474	0.13	1.94E-15	IGFBP5+
CITED2	9.74E-20	0.695546675	0.991	0.952	3.28E-15	IGFBP5+
PPP1R12B	2.08E-19	0.533237312	0.886	0.568	7.01E-15	IGFBP5+
MYH11	2.26E-19	0.422380547	0.474	0.139	7.61E-15	IGFBP5+
SLC20A2	2.57E-19	0.407891427	0.702	0.356	8.65E-15	IGFBP5+
ABLIM1	4.78E-19	0.319830076	0.667	0.301	1.61E-14	IGFBP5+
RBP1	5.64E-19	0.44221434	1	1	1.90E-14	IGFBP5+
MYH9	5.88E-19	0.483908342	0.982	0.934	1.98E-14	IGFBP5+
H3F3A	1.02E-18	0.392084309	0.982	0.996	3.44E-14	IGFBP5+
DDB1	1.11E-18	0.469023914	0.93	0.776	3.73E-14	IGFBP5+
SERPINH1	1.16E-18	0.471768796	1	0.92	3.90E-14	IGFBP5+
MYC	1.31E-18	0.836290383	0.842	0.649	4.41E-14	IGFBP5+
MARCKS	1.70E-18	0.452564156	0.947	0.867	5.72E-14	IGFBP5+
YPEL1	3.63E-18	0.259095501	0.544	0.19	1.22E-13	IGFBP5+
SMAD3	6.29E-18	0.42055017	0.851	0.598	2.12E-13	IGFBP5+
RAB31	7.43E-18	0.434309275	0.939	0.802	2.50E-13	IGFBP5+
TCEA3	8.58E-18	0.302673462	0.544	0.201	2.89E-13	IGFBP5+
WISP2	9.76E-18	0.649412508	0.737	0.422	3.29E-13	IGFBP5+

SEPP1	1.03E-17	1.05202486	0.904	0.819	3.48E-13	IGFBP5+
JADE1	1.34E-17	0.768123276	0.921	0.799	4.53E-13	IGFBP5+
SFRP1	1.64E-17	0.554409747	0.456	0.146	5.53E-13	IGFBP5+
HDAC2	1.98E-17	0.47782492	0.912	0.84	6.68E-13	IGFBP5+
SPARCL1	2.67E-17	0.43070056	0.474	0.151	9.01E-13	IGFBP5+
SLC4A7	2.80E-17	0.367068295	0.649	0.319	9.44E-13	IGFBP5+
RPSA	3.33E-17	0.298921396	1	0.993	1.12E-12	IGFBP5+
RP4-621F18.2	3.33E-17	0.312510524	0.404	0.103	1.12E-12	IGFBP5+
SEMA7A	6.57E-17	0.27565187	0.719	0.347	2.21E-12	IGFBP5+
ARID5B	7.95E-17	0.677390464	0.921	0.842	2.68E-12	IGFBP5+
HEG1	8.70E-17	0.477341423	0.711	0.42	2.93E-12	IGFBP5+
MRPL15	4.82E-16	0.347955721	0.851	0.674	1.62E-11	IGFBP5+
SIGLEC11	5.32E-16	0.334286836	0.632	0.288	1.79E-11	IGFBP5+
GSTA1	6.71E-16	0.601415412	0.974	0.87	2.26E-11	IGFBP5+
NDRG2	9.42E-16	0.384124087	0.825	0.544	3.17E-11	IGFBP5+
ARL6IP5	1.10E-15	0.666435368	0.965	0.957	3.70E-11	IGFBP5+
YBX3	1.26E-15	0.480908987	0.965	0.966	4.25E-11	IGFBP5+
SLC40A1	1.31E-15	0.527101972	0.509	0.205	4.42E-11	IGFBP5+
ABCA8	2.09E-15	0.61640469	0.772	0.464	7.03E-11	IGFBP5+
UGCG	2.12E-15	0.542638671	0.667	0.374	7.15E-11	IGFBP5+
TXN	2.55E-15	0.400302983	0.939	0.943	8.60E-11	IGFBP5+
CEBPD	3.13E-15	0.610900238	0.833	0.665	1.05E-10	IGFBP5+
ADAMTS6	3.18E-15	0.393468179	0.421	0.13	1.07E-10	IGFBP5+
AMIGO2	3.21E-15	0.532630804	0.667	0.379	1.08E-10	IGFBP5+
EIF4A1	4.20E-15	0.35410304	0.974	0.984	1.42E-10	IGFBP5+
SERINC5	4.62E-15	0.288327985	0.596	0.269	1.56E-10	IGFBP5+
SLITRK4	6.82E-15	0.321431217	0.421	0.13	2.30E-10	IGFBP5+
DBN1	8.35E-15	0.35896814	0.868	0.649	2.81E-10	IGFBP5+
GBP2	9.50E-15	0.351406873	0.632	0.315	3.20E-10	IGFBP5+
LIMS1	1.21E-14	0.449961733	0.912	0.861	4.08E-10	IGFBP5+
APEX1	1.45E-14	0.332210744	0.912	0.849	4.89E-10	IGFBP5+
UCHL1	2.34E-14	0.433162878	0.842	0.674	7.90E-10	IGFBP5+
RBMX	2.46E-14	0.371434032	0.93	0.931	8.28E-10	IGFBP5+
METAP2	3.08E-14	0.266742569	0.965	0.907	1.04E-09	IGFBP5+
FAM60A	4.95E-14	0.338480276	0.781	0.55	1.67E-09	IGFBP5+
NOP56	5.46E-14	0.343801944	0.868	0.678	1.84E-09	IGFBP5+

NPC2	6.38E-14	0.418076515	0.965	0.957	2.15E-09	IGFBP5+
CADM4	7.14E-14	0.276627642	0.728	0.413	2.41E-09	IGFBP5+
CCDC74A	8.70E-14	0.403932941	0.693	0.427	2.93E-09	IGFBP5+
GPX3	1.06E-13	0.506836406	0.588	0.286	3.58E-09	IGFBP5+
FN1	1.60E-13	0.751478574	1	1	5.40E-09	IGFBP5+
TXNRD1	1.77E-13	0.461749446	0.982	0.945	5.97E-09	IGFBP5+
TMEM245	1.86E-13	0.385944832	0.895	0.715	6.27E-09	IGFBP5+
CSDC2	1.94E-13	0.307822632	0.912	0.747	6.55E-09	IGFBP5+
KRT8	2.49E-13	0.676270541	0.772	0.614	8.39E-09	IGFBP5+
FRMD4B	3.06E-13	0.283808679	0.658	0.359	1.03E-08	IGFBP5+
PEG10	3.18E-13	0.392223705	0.649	0.345	1.07E-08	IGFBP5+
IQCG	4.83E-13	0.35453257	0.868	0.753	1.63E-08	IGFBP5+
LXN	5.27E-13	0.444804556	0.781	0.571	1.78E-08	IGFBP5+
OLA1	5.78E-13	0.289248274	0.877	0.785	1.95E-08	IGFBP5+
NPM1	7.27E-13	0.320843399	0.982	0.988	2.45E-08	IGFBP5+
NFE2L2	8.37E-13	0.327355928	0.956	0.895	2.82E-08	IGFBP5+
TMEM98	9.98E-13	0.30555599	0.868	0.74	3.36E-08	IGFBP5+
RPAIN	1.04E-12	0.327062408	0.851	0.799	3.51E-08	IGFBP5+
NDFIP1	1.17E-12	0.532451899	0.974	0.986	3.95E-08	IGFBP5+
S100A13	1.47E-12	0.444488893	0.965	0.963	4.96E-08	IGFBP5+
FSCN1	1.63E-12	0.464168092	0.904	0.77	5.49E-08	IGFBP5+
NBL1	1.85E-12	0.439467001	0.798	0.6	6.25E-08	IGFBP5+
WLS	1.87E-12	0.623889342	0.614	0.359	6.31E-08	IGFBP5+
LAPTM4B	2.10E-12	0.339545572	0.877	0.806	7.07E-08	IGFBP5+
DDAH1	2.77E-12	0.290406533	0.667	0.39	9.34E-08	IGFBP5+
MRAS	3.27E-12	0.291888921	0.623	0.359	1.10E-07	IGFBP5+
PCBP2	3.46E-12	0.264376524	0.974	0.988	1.17E-07	IGFBP5+
SNAPC1	3.51E-12	0.467114435	0.675	0.418	1.18E-07	IGFBP5+
RHOBTB3	3.73E-12	0.630515213	1	0.98	1.26E-07	IGFBP5+
CALD1	3.81E-12	0.541846592	0.921	0.877	1.28E-07	IGFBP5+
KRT18	4.05E-12	0.819627948	0.649	0.416	1.36E-07	IGFBP5+
GNL3	4.71E-12	0.286298965	0.825	0.594	1.59E-07	IGFBP5+
VCL	6.74E-12	0.418191858	1	0.97	2.27E-07	IGFBP5+
ADGRL2	8.19E-12	0.292643315	0.675	0.39	2.76E-07	IGFBP5+
C1orf141	1.04E-11	0.361257848	0.754	0.482	3.49E-07	IGFBP5+
LCMT1	1.24E-11	0.334299332	0.974	0.959	4.19E-07	IGFBP5+

RP11-347P5.1	1.28E-11	0.499401409	0.526	0.247	4.32E-07	IGFBP5+
SEC14L1	1.58E-11	0.281143181	0.912	0.815	5.33E-07	IGFBP5+
DDX5	1.68E-11	0.255492663	1	0.996	5.64E-07	IGFBP5+
OXTR	1.80E-11	0.348051464	0.763	0.445	6.07E-07	IGFBP5+
GPX4	2.33E-11	0.310573046	0.956	0.98	7.85E-07	IGFBP5+
HN1	2.46E-11	0.426256994	0.921	0.897	8.28E-07	IGFBP5+
ENC1	2.49E-11	0.255360412	0.298	0.084	8.39E-07	IGFBP5+
ERRFI1	2.51E-11	0.5428947	0.912	0.843	8.47E-07	IGFBP5+
MFGE8	2.57E-11	0.292972663	0.868	0.685	8.68E-07	IGFBP5+
TKT	3.02E-11	0.300570158	0.912	0.881	1.02E-06	IGFBP5+
HUWE1	3.53E-11	0.41632007	0.868	0.708	1.19E-06	IGFBP5+
TAGLN	4.07E-11	0.608597111	0.553	0.299	1.37E-06	IGFBP5+
SMARCC1	5.37E-11	0.293103106	0.711	0.539	1.81E-06	IGFBP5+
NEAT1	5.67E-11	0.480719643	0.991	0.998	1.91E-06	IGFBP5+
KHDRBS1	6.34E-11	0.31696543	0.93	0.907	2.13E-06	IGFBP5+
RRAD	6.39E-11	0.918140328	0.64	0.415	2.15E-06	IGFBP5+
SYNE2	6.56E-11	0.332869735	0.719	0.468	2.21E-06	IGFBP5+
C19orf48	7.61E-11	0.368670379	0.772	0.633	2.56E-06	IGFBP5+
PFDN4	1.03E-10	0.295892469	0.851	0.845	3.47E-06	IGFBP5+
CDH2	1.14E-10	0.317884611	0.974	0.945	3.83E-06	IGFBP5+
SERPINE2	1.24E-10	0.869339743	1	0.984	4.16E-06	IGFBP5+
GTF2I	1.39E-10	0.292749503	0.895	0.819	4.69E-06	IGFBP5+
ATP1B3	1.58E-10	0.3156266	0.947	0.849	5.34E-06	IGFBP5+
HNRNPA0	1.59E-10	0.305649491	0.956	0.954	5.35E-06	IGFBP5+
ATRX	1.59E-10	0.356858291	0.947	0.899	5.36E-06	IGFBP5+
ILF2	1.86E-10	0.266185904	0.93	0.883	6.26E-06	IGFBP5+
PIM1	1.86E-10	0.268317801	0.561	0.304	6.27E-06	IGFBP5+
PUM3	1.88E-10	0.259409803	0.675	0.42	6.35E-06	IGFBP5+
TRIM28	2.29E-10	0.262541586	0.86	0.637	7.73E-06	IGFBP5+
CDK5RAP2	2.50E-10	0.752648237	0.711	0.53	8.42E-06	IGFBP5+
TBC1D23	3.25E-10	0.367851709	0.833	0.714	1.09E-05	IGFBP5+
GSN	3.51E-10	0.260492078	0.842	0.71	1.18E-05	IGFBP5+
MYL9	4.58E-10	0.402627549	0.965	0.954	1.54E-05	IGFBP5+
CTSC	5.20E-10	0.298307279	0.632	0.359	1.75E-05	IGFBP5+
CTGF	7.22E-10	0.572797971	0.605	0.352	2.43E-05	IGFBP5+
GREM1	8.54E-10	0.704182558	0.377	0.164	2.88E-05	IGFBP5+

TMSB4X	9.99E-10	0.324623496	1	1	3.37E-05	IGFBP5+
MAP2K3	1.02E-09	0.256615501	0.746	0.537	3.43E-05	IGFBP5+
ZFAS1	1.52E-09	0.292312515	0.895	0.913	5.13E-05	IGFBP5+
PAWR	1.75E-09	0.254070681	0.754	0.546	5.90E-05	IGFBP5+
PRSS23	1.97E-09	0.452689441	0.982	0.934	6.65E-05	IGFBP5+
MAGED2	2.22E-09	0.365856994	0.991	0.977	7.49E-05	IGFBP5+
GNPTAB	2.24E-09	0.292341797	0.833	0.68	7.56E-05	IGFBP5+
NAV2	2.28E-09	0.275049193	0.605	0.34	7.67E-05	IGFBP5+
COL1A1	2.77E-09	0.613840028	0.579	0.356	9.34E-05	IGFBP5+
OCIAD2	2.88E-09	0.264106449	0.816	0.73	9.70E-05	IGFBP5+
CFI	2.95E-09	0.284537389	0.86	0.717	9.96E-05	IGFBP5+
KTN1	3.30E-09	0.2882101	1	0.977	0.000111276	IGFBP5+
ZNF106	3.70E-09	0.260678078	0.86	0.71	0.000124816	IGFBP5+
AKAP9	3.94E-09	0.280742959	0.956	0.943	0.00013287	IGFBP5+
SYTL4	4.27E-09	0.34463899	0.658	0.436	0.000143761	IGFBP5+
EIF5B	4.91E-09	0.252391874	0.93	0.902	0.000165593	IGFBP5+
BGN	6.05E-09	0.296717074	0.579	0.317	0.000203849	IGFBP5+
IGF1R	6.45E-09	0.320058804	0.93	0.84	0.000217252	IGFBP5+
SLC38A2	1.43E-08	0.342202417	0.965	0.923	0.000483223	IGFBP5+
HMGA2	1.51E-08	0.315454719	0.509	0.306	0.000507416	IGFBP5+
HMGN5	2.20E-08	0.428005486	0.649	0.411	0.000742794	IGFBP5+
HMGB1	2.72E-08	0.343057595	0.982	0.973	0.000915859	IGFBP5+
IER3	3.17E-08	0.405671686	0.956	0.954	0.001067861	IGFBP5+
PBX1	3.18E-08	0.255972682	0.658	0.445	0.001071878	IGFBP5+
TIMP3	3.26E-08	0.813418536	0.632	0.438	0.001099893	IGFBP5+
GRB14	3.60E-08	0.389640834	0.754	0.593	0.001213354	IGFBP5+
NCL	3.84E-08	0.252606652	0.956	0.94	0.001295075	IGFBP5+
TBC1D4	4.14E-08	0.271921042	0.596	0.377	0.001396045	IGFBP5+
ZNF281	4.52E-08	0.278655187	0.711	0.543	0.00152351	IGFBP5+
EPS8	5.26E-08	0.320421138	0.912	0.875	0.001770844	IGFBP5+
DSP	6.17E-08	0.304756919	0.781	0.598	0.002080457	IGFBP5+
GSTA4	6.47E-08	0.317170228	0.947	0.893	0.002181226	IGFBP5+
TMEM200A	7.19E-08	0.309932611	0.789	0.628	0.002423401	IGFBP5+
AXL	8.18E-08	0.296515085	0.982	0.922	0.002757465	IGFBP5+
PEG3	9.52E-08	0.326736909	0.728	0.489	0.003208429	IGFBP5+
ENAH	1.02E-07	0.35148365	0.974	0.957	0.003421114	IGFBP5+

IGFBP1	1.07E-07	0.72989594	0.325	0.137	0.003603141	IGFBP5+
CTSA	1.11E-07	0.278614734	0.912	0.792	0.003739616	IGFBP5+
TSPAN6	1.33E-07	0.303214986	0.781	0.617	0.004483472	IGFBP5+
LACTB	1.33E-07	0.260111215	0.816	0.701	0.004493341	IGFBP5+
JARID2	1.55E-07	0.270472682	0.658	0.445	0.005233026	IGFBP5+
STAMBPL1	1.69E-07	0.38578397	0.553	0.365	0.005709664	IGFBP5+
MLLT11	2.00E-07	0.324031047	0.5	0.302	0.006748567	IGFBP5+
SRGAP1	2.01E-07	0.273157492	0.789	0.642	0.006776784	IGFBP5+
PRKCA	2.25E-07	0.327757316	0.956	0.897	0.007574925	IGFBP5+
SNHG8	2.80E-07	0.269489631	0.816	0.731	0.009449018	IGFBP5+

Control 2 (C2)

p_value is from the negantive-binomial test for the detection differential genes over the two cell groups.
avg_logFC is for the average logorithm values of fold difference.
pct.1 is for the percentage of cells that gives the signal on the designate gene on the target cell group/cluster.
pct.2 is for the percentage of cells that give the signal on the designate gene on the counterpart of the target cell group/cluster.
p_val_adj is the p value after benferroni correction of multi-tests study.

gene name	p_val	avg_logFC	pct.1	pct.2	p_val_adj	Cluster_Ids
GJA1	6.75E-26	0.472451649	0.996	0.9	2.27E-21	IGFBP5+
RDX	3.12E-23	0.47189783	0.996	0.935	1.05E-18	IGFBP5+
BEX1	5.42E-22	0.55025875	0.968	0.787	1.83E-17	IGFBP5+
SPARC	8.23E-21	0.349897272	1	0.976	2.77E-16	IGFBP5+
TMEM123	1.01E-16	0.409454828	0.855	0.522	3.39E-12	IGFBP5+
GSTA1	7.07E-16	0.652265673	0.783	0.505	2.38E-11	IGFBP5+
GSTA4	1.18E-13	0.363068644	0.707	0.411	3.97E-09	IGFBP5+
NUPR1	1.36E-13	0.473175666	0.759	0.455	4.59E-09	IGFBP5+
RAB31	2.88E-13	0.412832282	0.755	0.471	9.69E-09	IGFBP5+
RNASE1	3.96E-13	0.273329685	0.345	0.112	1.33E-08	IGFBP5+
ULBP1	4.23E-13	0.441201622	0.719	0.445	1.42E-08	IGFBP5+
IGFBP5	5.00E-13	0.614377784	0.96	0.792	1.68E-08	IGFBP5+
CXXC5	6.65E-13	0.341292016	0.602	0.325	2.24E-08	IGFBP5+
CALB2	1.96E-12	0.376850257	0.667	0.378	6.60E-08	IGFBP5+
LRAT	2.12E-12	0.265366669	0.767	0.476	7.12E-08	IGFBP5+
GBP2	4.46E-12	0.420376722	0.422	0.184	1.50E-07	IGFBP5+
DAAM1	6.49E-12	0.276348223	0.687	0.411	2.18E-07	IGFBP5+
SIGLEC11	7.71E-12	0.26357335	0.454	0.201	2.60E-07	IGFBP5+
LMO4	1.85E-11	0.370658359	0.506	0.258	6.24E-07	IGFBP5+
CDH11	3.01E-11	0.389202561	0.566	0.309	1.01E-06	IGFBP5+
FSCN1	3.54E-11	0.312730448	0.735	0.49	1.19E-06	IGFBP5+
STK17B	3.79E-11	0.396147512	0.494	0.251	1.27E-06	IGFBP5+
LRRFIP2	7.07E-11	0.331400046	0.799	0.533	2.38E-06	IGFBP5+
TNNI3	7.88E-11	0.384547542	0.414	0.184	2.65E-06	IGFBP5+
ITGA1	8.98E-11	0.33890407	0.333	0.124	3.02E-06	IGFBP5+
S100A16	1.21E-10	0.270188853	0.831	0.584	4.08E-06	IGFBP5+
TIMP3	1.26E-10	0.330574909	0.723	0.457	4.23E-06	IGFBP5+
FAM198B	1.79E-10	0.334060915	0.594	0.337	6.01E-06	IGFBP5+

MEF2C	2.43E-10	0.278846705	0.574	0.323	8.19E-06	IGFBP5+
HMGB1	3.06E-10	0.33694414	0.815	0.586	1.03E-05	IGFBP5+
SMS	5.59E-10	0.261400107	0.618	0.371	1.88E-05	IGFBP5+
TNC	1.11E-09	0.367162666	0.936	0.782	3.74E-05	IGFBP5+
RPS27L	1.57E-09	0.329326953	0.827	0.603	5.28E-05	IGFBP5+
THBS1	1.62E-09	0.444933067	0.839	0.596	5.47E-05	IGFBP5+
S100A13	2.02E-09	0.364911523	0.723	0.517	6.79E-05	IGFBP5+
PPP1R12B	2.82E-09	0.274156443	0.534	0.297	9.48E-05	IGFBP5+
SOX4	3.17E-09	0.656525915	0.494	0.292	0.00010674	IGFBP5+
PRKCA	5.78E-09	0.277906671	0.703	0.467	0.000194685	IGFBP5+
MRAS	6.73E-09	0.287109293	0.285	0.115	0.00022654	IGFBP5+
BOD1	6.83E-09	0.300129425	0.369	0.177	0.00022998	IGFBP5+
CBLB	7.57E-09	0.255632467	0.622	0.373	0.000254832	IGFBP5+
GPRC5B	8.84E-09	0.330422069	0.293	0.122	0.000297509	IGFBP5+
TOMM7	1.24E-08	0.274680179	0.691	0.45	0.000418157	IGFBP5+
FBXL20	1.28E-08	0.266900973	0.574	0.34	0.000431513	IGFBP5+
CTNNAL1	1.37E-08	0.256781657	0.835	0.629	0.000460672	IGFBP5+
PEG3	1.37E-08	0.259933452	0.691	0.459	0.000462665	IGFBP5+
OXTR	1.51E-08	0.313950744	0.51	0.289	0.000509836	IGFBP5+
EPS8	3.63E-08	0.290492582	0.518	0.311	0.001222839	IGFBP5+
FABP3	4.09E-08	0.343553608	0.618	0.414	0.001376986	IGFBP5+
GNAI1	5.21E-08	0.311421189	0.426	0.23	0.001754462	IGFBP5+
GMNN	5.36E-08	0.461877666	0.249	0.098	0.001803231	IGFBP5+
TJP1	7.99E-08	0.271504594	0.382	0.194	0.002689868	IGFBP5+
WFDC3	8.11E-08	0.300350744	0.289	0.122	0.002729653	IGFBP5+
PNCK	9.98E-08	0.271473413	0.659	0.447	0.003360632	IGFBP5+
PSD3	1.15E-07	0.29500892	0.606	0.392	0.003879878	IGFBP5+
NDRG2	1.18E-07	0.261741213	0.43	0.242	0.003984253	IGFBP5+
OTOF	1.36E-07	0.320416449	0.269	0.117	0.004577452	IGFBP5+
NDFIP1	2.28E-07	0.372610825	0.687	0.517	0.007678562	IGFBP5+
BEX2	2.54E-07	0.250317928	0.534	0.325	0.008561216	IGFBP5+
MAP1LC3A	2.80E-07	0.284696159	0.635	0.423	0.009441373	IGFBP5+
