# Supplementary material for: CD24: a marker of granulosa cell subpopulation and a mediator of ovulation
Source: Cell Death Dis. 2019 Oct 17;10(11):791. doi: 10.1038/s41419-019-1995-1 (PMC6797718; doi:10.1038/s41419-019-1995-1)
Supplement: Supplementary file 7 — Supplementary Table S4 Results of pathway analysis using REACTOME [file 41419_2019_1995_MOESM7_ESM.docx]

Supplementary Table S4. Results of pathway analysis using REACTOME

| **ID** | **GeneRatio** | **BgRatio** | **pvalue** | **p.adjust** | **qvalue** | **geneID** | **Count** |
| --- | --- | --- | --- | --- | --- | --- | --- |
| ***Gene set 1 (combined gene set 1 of C1 and C2)*** | | | | | | | |
| REACTOME_METABOLISM_OF_LIPIDS_AND_  LIPOPROTEINS | 18/96 | 478/6025 | 0.000453932 | 0.029695378 | 0.0290349 | HSD11B1/PEMT/P4HB/HSD17B12/DHCR24/DHCR7/PSAP/PLIN2/FDPS/MSMO1/FABP6/IDI1/  LPCAT1/SQLE/NSDHL/MVD/GBA2/HSD17B1 | 18 |
| REACTOME_DIABETES_  PATHWAYS | 9/96 | 133/6025 | 0.000242467 | 0.028974774 | 0.028330323 | PDIA5/HSPA5/DNAJB9/SSR1/PDIA6/SPCS2/HSP90B1/IGFBP4/  IGFBP2 | 9 |
| REACTOME_CHOLESTEROL_BIOSYNTHESIS | 8/96 | 24/6025 | 1.85E-09 | 4.41E-07 | 4.31E-07 | DHCR24/DHCR7/FDPS/MSMO1/IDI1/SQLE/NSDHL/MVD | 8 |
| REACTOME_RESPONSE_TO_ELEVATED_PLATELET_CYTOSOLIC_CA2_ | 7/96 | 89/6025 | 0.000496994 | 0.029695378 | 0.0290349 | SRGN/CAP1/PSAP/HSPA5/CLU/CD63/ACTN2 | 7 |
|  | | | | | | | |
| ***Gene set 3 (combined gene set 3 of C1 and C2)*** | | | | | | | |
| REACTOME_DEVELOPMENTAL_BIOLOGY | 19/95 | 396/6025 | 9.66744E-06 | 0.000841067 | 0.000790356 | COL1A2/GDNF/COL3A1/NTN4/NRP2/MYH11/ABLIM1/MYH9/SMAD3/SEMA7A/CEBPD/CDH2/MYL9/COL1A1/ENAH/  SRGAP1/RDX/ITGA1/MEF2C | 19 |
| REACTOME_AXON_  GUIDANCE | 15/95 | 251/6025 | 7.21868E-06 | 0.000841067 | 0.000790356 | COL1A2/GDNF/COL3A1/NTN4/NRP2/MYH11/ABLIM1/MYH9/SEMA7A/MYL9/COL1A1/ENAH/SRGAP1/RDX/ITGA1 | 15 |
| REACTOME_MUSCLE_  CONTRACTION | 7/95 | 48/6025 | 8.42689E-06 | 0.000841067 | 0.000790356 | VIM/MYH11/CALD1/VCL/MYL9/TNNI3/ITGA1 | 7 |
| REACTOME_SMOOTH_MUSCLE_CONTRACTION | 5/95 | 25/6025 | 3.62895E-05 | 0.00236789 | 0.00222512 | MYH11/CALD1/VCL/MYL9/  ITGA1 | 5 |
