# Supplementary material for: CD24: a marker of granulosa cell subpopulation and a mediator of ovulation
Source: Cell Death Dis. 2019 Oct 17;10(11):791. doi: 10.1038/s41419-019-1995-1 (PMC6797718; doi:10.1038/s41419-019-1995-1)
Supplement: Supplementary file 8 — MATERIALSupplementary Table S5. Clinical and biochemical profiles of woman with PCOS and control [file 41419_2019_1995_MOESM8_ESM.docx]

Supplementary Table S5. Clinical and biochemical profiles of woman with PCOS and control

| **Variable** | **Group 1 (n=2)** | | **Group 2 (n=14)** | | **Group 3 (n=10)** | |
| --- | --- | --- | --- | --- | --- | --- |
|  | **Control 1** | **Control 2** | **Control** | **PCOS** | **Control** | **PCOS** |
| No. | 1 | 1 | 7 | 7 | 5 | 5 |
| Age (years) | 26 | 28 | 28.14±0.99 | 27.14±1.8 | 28±1.90 | 28.6±0.49 |
| BMI (Kg/m2) | 20.02 | 19.30 | 20.2±1.21 | 21.9±1.62 | 21.26±0.63 | 21.2±2.98 |
| Basal LH(IU/L) | 4.05 | 4.22 | 3.72±0.27 | 7.54±1.62 | 2.64±0.86 | 5.19±2.59 |
| Basal FSH(IU/L) | 5.43 | 5.51 | 5.83±1.00 | 4.65±0.60 | 5.05±1.52 | 5.83±1.46 |
| Basal T (ng/dL) | 29.31 | 31.69 | 31.17±10.05 | 45.68±7.57 | 22.70±4.54 | 47.24±8.58 |
| AMH (ng/mL) | 4.65 | 3.72 | 2.72±0.67 | 12.84±5.97 | 3.80±1.01 | 7.95±0.93 |
| Hormones on hCG day E2(pg/mL) | 3048 | 2656 | 3427.43±736.52 | 6506.86±1739.62 | 3342±682.24 | 5296±2108.35 |
