# Supplementary material for: CD24: a marker of granulosa cell subpopulation and a mediator of ovulation
Source: Cell Death Dis. 2019 Oct 17;10(11):791. doi: 10.1038/s41419-019-1995-1 (PMC6797718; doi:10.1038/s41419-019-1995-1)
Supplement: Supplementary file 9 — Supplementary Table S6. Primer sequences used for real-time PCR analyses [file 41419_2019_1995_MOESM9_ESM.docx]

Supplementary Table S6. Primer sequences used for real-time PCR analyses

| **Genes** | **Accession no** | **Forward primers** | **Reverse primers** |
| --- | --- | --- | --- |
| **Human** |  |  |  |
| *CD24* | [NM_013230](http://www.ncbi.nlm.nih.gov/entrez/query.fcgi?cmd=Search&db=Nucleotide&term=NM_013230) | CTCCTACCCACGCAGATTTATTC | AGAGTGAGACCACGAAGAGAC |
| *PTGES* | NM_004878 | GCTGATCACACCCACAGTTG | CCAGGAAAAGGAAGGGGTAG |
| *PTGS2* | NM_000963 | TGAAACCCACTCCAAACACA | GAGAAGGCTTCCCAGCTTTT |
| *AKR1C1* | NM_001353 | ATTCCCATCGACCAGAGTTG | CATGTGGCACAGAGATCCAC |
| *ABCC4* | NM_005845 | GGCTTGTGCTCTGAAAAAGG | CTGAGAGGATCGTCCAGGAG |
| *PLA2G4A* | NM_024420 | AACCAAGTGCCTGTGGTAGC | GGCCCTTTCTCTGGAAAATC |
| *SLCO2A1* | NM_005630 | TCAACATGAGCTCTGCAACC | ATCAACAAGAACTGCACCCC |
| *GAPDH* | [NM_001256799](http://www.ncbi.nlm.nih.gov/entrez/query.fcgi?cmd=Search&db=Nucleotide&term=NM_001256799) | CGGATTTGGTCGTATTGGG | CTGGAAGATGGTGATGGGATT |
| **Mouse** |  |  |  |
| *Cd24a* | NM_009846 | ACATCTAGAGAGTCGCGCCG | ACGGTGCAACAGATGTTTGG |
| *Ptges* | [NM_022415](http://www.ncbi.nlm.nih.gov/entrez/query.fcgi?cmd=Search&db=Nucleotide&term=NM_022415) | CACACTGCTGGTCATCAAGAT | TCACTCCTGTAATACTGGAGGC |
| *Ptgs2* | [NM_011198](http://www.ncbi.nlm.nih.gov/entrez/query.fcgi?cmd=Search&db=Nucleotide&term=NM_011198) | TTCAACACACTCTATCACTGGC | AGAAGCGTTTGCGGTACTCAT |
| *Akr1c1* | NM_030611 | CCTGTGTGCAACCAGGTAGA | CCCATTGTTTTTCACGATGGCT |
| *Abcc4* | [NM_001033336](http://www.ncbi.nlm.nih.gov/entrez/query.fcgi?cmd=Search&db=Nucleotide&term=NM_001033336) | CATCGCGGTAACCGTCCTC | CCGCAGTTTTACTCCGCAG |
| *Pla2g4a* | [NM_008869](http://www.ncbi.nlm.nih.gov/entrez/query.fcgi?cmd=Search&db=Nucleotide&term=NM_008869) | CAGCACATTATAGTGGAACACCA | AGTGTCCAGCATATCGCCAAA |
| *Slco2a1* | [NM_033314](http://www.ncbi.nlm.nih.gov/entrez/query.fcgi?cmd=Search&db=Nucleotide&term=NM_033314) | TGAAGCGTTTTGTTTTCCCTCT | CGGGTGTGGAACATCCCATAA |
| *Gapdh* | [NM_008084](http://www.ncbi.nlm.nih.gov/entrez/query.fcgi?cmd=Search&db=Nucleotide&term=NM_008084) | AGGTCGGTGTGAACGGATTTG | TGTAGACCATGTAGTTGAGGTCA |
